# Role of Citrate in Pathophysiology and Medical Management of Bone Diseases

**DOI:** 10.3390/nu11112576

**Published:** 2019-10-25

**Authors:** Donatella Granchi, Nicola Baldini, Fabio Massimo Ulivieri, Renata Caudarella

**Affiliations:** 1 Laboratory for Orthopedic Pathophysiology and Regenerative Medicine, IRCCS Istituto Ortopedico Rizzoli, via di Barbiano 1/10, 40136 Bologna, Italy; nicola.baldini@ior.it; 2 Department of Biomedical and Neuromotor Sciences, Via Pupilli 1, University of Bologna, 40136 Bologna, Italy; 3 Nuclear Medicine, Bone Metabolic Unit, IRCCS Ca’ Granda Ospedale Maggiore Policlinico, Via F.Sforza 35, 20122 Milano, Italy; ulivieri@gmail.com; 4 Maria Cecilia Hospital, GVM Care and Research, Via Corriera 1, 48033 Cotignola (RA), Italy; renata.caudarella@gmail.com

**Keywords:** bone metabolism, bone mineral density, bone remodelling, citrate supplement, osteopenia, osteoporosis, kidney diseases

## Abstract

Citrate is an intermediate in the “Tricarboxylic Acid Cycle” and is used by all aerobic organisms to produce usable chemical energy. It is a derivative of citric acid, a weak organic acid which can be introduced with diet since it naturally exists in a variety of fruits and vegetables, and can be consumed as a dietary supplement. The close association between this compound and bone was pointed out for the first time by Dickens in 1941, who showed that approximately 90% of the citrate bulk of the human body resides in mineralised tissues. Since then, the number of published articles has increased exponentially, and considerable progress in understanding how citrate is involved in bone metabolism has been made. This review summarises current knowledge regarding the role of citrate in the pathophysiology and medical management of bone disorders.

## 1. Introduction

Citrate is an intermediate in the tricarboxylic acid cycle (TCA cycle, Krebs cycle), a central metabolic pathway for all aerobic organisms, including animals, plants, and bacteria [1,2]. In humans, citrate is produced in the mitochondria after the condensation of acetyl coenzyme A (acetylCoA) and oxaloacetate, which are catalysed by citrate synthase; it then enters the TCA cycle, thus becoming the primary adenosine 5′-triphosphate (ATP) provider by which living cells harvest the energy they need to accomplish essential and specific functions [1].

**Figure 1:**
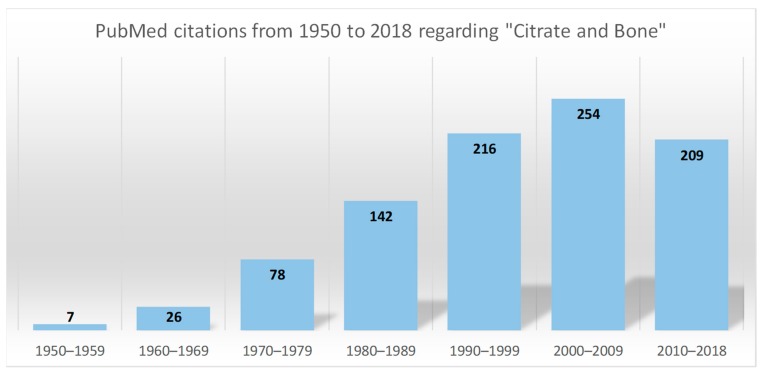
Distribution of the biomedical citations indexed by PubMed over seven decades (*n* = 949, from 1949 to 2018). The search query focused on “citrate” and “bone”, with a search restricted to the terms “Title”, “Abstract” or “Medical Subject Headings”. Only citations related to studies on humans are included with the exception of those dealing with citrate as an anticoagulant.

The intracellular citrate level reflects the energy status of the cell and acts as a regulator. When the cellular ATP is abundant and the energy demand of the cells is low, the excess citrate can be exported to the cytosol by means of a mitochondrial citrate carrier [3]. It can be used for the lipid biosynthesis of highly proliferating cells [4] or for supporting the tissue-related functions of specialised cells, including the mineralisation of the extracellular matrix by osteoblasts, the bone-forming cells [5]. In this regard, the close association between citrate and bone was pointed out for the first time by Dickens in 1941 [6]. Since then, the number of published articles dealing with this topic has increased exponentially (Figure 1), and considerable progress in understanding how citrate is involved in bone metabolism has been made. This review summarises the current knowledge regarding the relationship between citrate and bone pathophysiology, starting from the link with bone cells and the mineralised matrix, moving through the role of citrate in the onset of bone disorders, and concluding with a critical evaluation of the clinical use of citrate supplements for the medical management of bone diseases.

## 2. Citrate Homeostasis: General Physiological Concepts

### 2.1. The Pillars of Citrate Homeostasis

**Figure 2:**
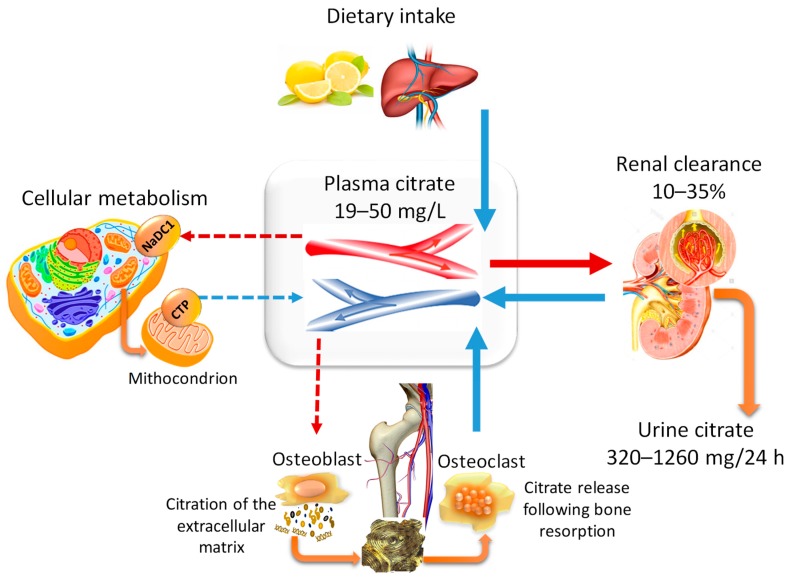
The four domains of citrate homeostasis. The plasma level of citrate mainly depends on four sources, i.e., nutritional intake, renal clearance, cellular metabolism, and bone remodelling. Food citrate is rapidly introduced into the circulation, filtered at the glomerular level, and eventually reabsorbed according to physiological needs. The citrate uptake from the extracellular milieu may occur only when specific transporter proteins are expressed, i.e., sodium-dicarboxylate (NaDC)1 belonging to the “solute carrier” 13 (Slc13) family. The citrate produced by mitochondria only marginally contributes to citrate homeostasis, since it is almost all used by cells as an energy source, or for the synthesis of lipids and other specific functions, i.e., citration of the extracellular matrix by the osteoblasts. In fact, the bulk stored in bone is the main endogenous source of citrate which becomes available following the resorption of the mineralised matrix by the osteoclasts. The mitochondrial citrate-transport protein (CTP) is essential for the release of citrate from the mitochondria to cytosol.

There is a balance between citrate availability and elimination, depending on physiological requirements. Basically, citrate homeostasis depends on four domains, i.e., nutritional intake, renal clearance, cellular metabolism, and bone remodelling (Figure 2).

Citric acid is naturally contained in fruits and vegetables, particularly in citrus fruits, with concentrations ranging from 0.005 mol/L in oranges and grapefruit to 0.30 mol/L in lemons and limes [7,8]. Food citrate may also be produced biotechnologically by many microorganisms through a fermentation process, and of these, Aspergillum Niger has been recognised as the most efficient producer [9]. Industrial-scale citric acid is employed instead of fresh lemon juice in a variety of pre-prepared meals, and it is also used as an additive in foods and beverages since it acts as a preservative, acidity regulator, flavoring substance and emulsifying agent. In fact, due to the variety of applications, citric acid is the most consumed organic acid in the world, leading to high commercial interest as well as motivating scientists to discover new super-producing techniques [10].

The usual nutritional intake of citrate is approximately 4 grams per day [11] and more than 95% of it is absorbed in the small intestine [12] by means of the sodium-dicarboxylate (NaDC) cotransporter similar to that described in the kidney [13]. The total daily consumption of citric acid may exceed 500 mg/kg of body weight, but considering the low citrate amount in foods, both those in which it is naturally contained and those in which it is added in artificial form, the ingestion of excessive doses is very unlikely [14].

The dietary ingestion of citrate is able to enhance the plasma level within thirty minutes; it is then filtered at the glomerular level just as quickly, and eventually reabsorbed according to physiological needs [15]. In healthy individuals, the serum concentration of circulating citrate is relatively constant, ranging from 19 to 50 mg/L with an average of 20 mg/L [16,17]. Citrate in plasma exists as ≈95% tricarboxylate, ≈4% dicarboxylate, and 1% monocarboxylate, and the majority is complexed to divalent ions, such as calcium and magnesium. The high impermeability of the plasma membrane precludes the cellular uptake of citrate from the extracellular fluid. Under special conditions, some cells (i.e., intestinal enterocytes and kidney tubular cells) express a plasma membrane citrate transporter belonging to the “solute carrier” 13 family (Slc13, NaDC), which is essential for uptake from the gastrointestinal tract and tubular fluid.

However, the net balance between gastrointestinal absorption and the urinary excretion of citrate suggests that nutritional intake cannot be solely responsible for the maintenance of plasma homeostasis [15,17]. The cellular metabolism also has a scarce impact on citrate availability since almost all the mitochondrial production is consumed by cells as an energy source or for supporting specific cell functions [4,5]. Nowadays, it is well known that the main endogenous bulk of citrate is stored in bone and is mobilised following the resorption of the mineralised matrix by the osteoclasts [17].

### 2.2. Citraturia as A Marker of Citrate Homeostasis and Bone Health Status

As circulating citrate is freely filtered in the glomerulus, 24-h excretion is considered to be a valid marker for highlighting alterations of citrate homeostasis [11]. The reference values for urinary citrate levels range from 320 to 1260 mg/24 h, with an average in males of 550 mg/24 h and in females of 680 mg/24 h [18,19]. The higher excretion of citrate in females is in relation to the estrogenic rate [20] and explains the lower incidence of nephrolithiasis in premenopausal women, considering that citrate-calcium binding is one of the main mechanisms for inhibiting stone formation [21]. Based on the reference values for lithogenic risk, the threshold for the diagnosis of hypocitraturia is usually fixed as less than 320 mg per day. However, hypocitraturia may be severe (citrate excretion of less than 100 mg per day) or mild-moderate (from 100 to 320 mg), but low excretion (less than 640 mg per day) could also be a significant sign and should be monitored [18]. In general, elevated citrate excretion may be considered a non-pathological condition which reflects the restoration of the acid-base balance and occurs, for instance, after chronic alkali intake [22]. Low citrate excretion is a relatively common finding, and even though the majority of patients have idiopathic hypocitraturia, there are several medical and physiological conditions associated with this abnormality. All the conditions listed in Table 1 may be potentially associated with skeletal disorders or, more broadly, with bone metabolism alterations, even when there are no obvious symptoms.

Modulation of citrate excretion in the kidney is influenced by multiple factors, but pH regulation, particularly in the proximal tubule, has the strongest impact, and even a small decrease in tubular pH significantly increases tubular reabsorption [16]. Therefore, in response to the elevated acid load occurring in metabolic acidosis, there is a notable increase in citrate recovery with subsequent hypocitraturia; urinary citrate excretion may be used as a laboratory parameter for monitoring the diet- and metabolism-dependent systemic acid-base status, even in subjects without overt metabolic acidosis [29,35,36].

The detrimental effect of acid-base imbalance on bone metabolism has been proven without a doubt [36,37], thus suggesting that citraturia could be a noninvasive and indirect view of bone health status. Actually, the relationship among urinary citrate excretion, bone quality parameters, and circulating levels of bone turnover markers has been demonstrated [34,36], even if its clinical usefulness is still controversial [37].

## 3. Citrate and Bone Tissue

In 1941 Dickens stated that approximately 90% of the total citrate found in the body of “osteovertebrates” resides in mineralised tissues, but the most valuable insight was that, due to its high binding affinity to calcium stored in the hard tissue, citrate could play a pivotal role in regulating metabolic functions and in maintaining the structural integrity of bone [6]. Moreover, Dickens postulated that the presence of citrate in bone is crucial for preventing calcium precipitation, either when bone tissue is resorbed in response to lowered serum calcium or when the biomineralisation process starts. Over time, data in the literature regarding the relationship between citrate and bone physiology have been increasing exponentially (Figure 1), but the role of citrate in driving the structural and functional properties of healthy bone in humans has only been partially elucidated. Early studies were mainly aimed at searching for the origin and the role of calciotropic hormones (calcitonin, parathyroid hormone (PTH), and vitamin D) in the regulation of its metabolism. However, these issues have remained largely unresolved and/or highly speculative, due to the absence of necessary research methodology and technology. Nowadays, there is adequate knowledge regarding the role of bone cells in producing citrate, how citrate enters the crystalline structure of bone, and how it controls the size and morphology of apatite nanocrystals.

### 3.1. Citrate and Mineral Structure

**Figure 3:**
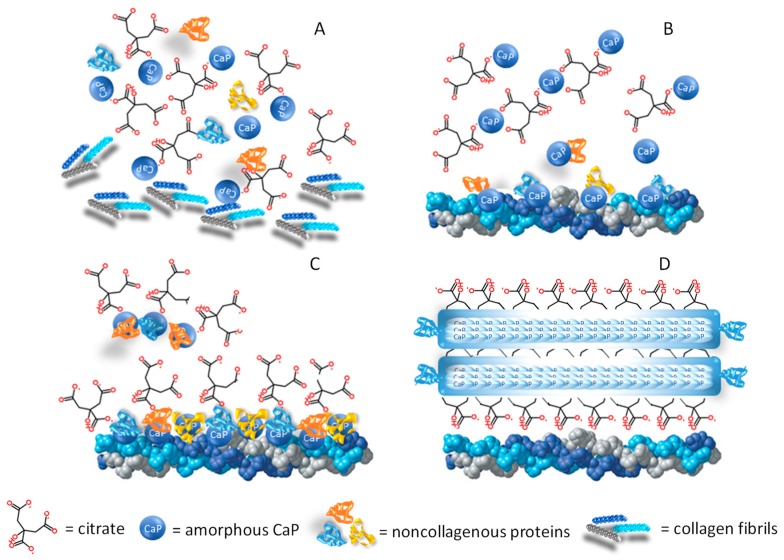
Citrate in the formation of the mineral matrix. The figure combines the theories proposed by different authors regarding the role of citrate in the mineralisation process [38,40,43,44,45]. (**A**) The amorphous calcium-phosphate (CaP) phase originates from an oversaturated CaP solution, and the mineralisation process starts when the organic phase (citrate, collagen fibrils, and noncollagenous proteins) is available in the bone microenvironment. (**B**) At the early stage, few citrate molecules bind with the amorphous CaP and the particle aggregation is slowed down. (**C**) In the next phase, the noncollagenous proteins released from bone cells favour CaP aggregation and apatite nucleation while the collagen promotes the self-assembly of CaP and guides the aggregate deposition on the collagen surface. (**D**) When the surface is fully covered by citrate, the thickness increase is inhibited (2–6 nm), while longitudinal growth continues up to 30–50 nm, thus explaining the flat morphology of bone mineral platelets. In addition, citrate forms bridges between the mineral platelets which can explain the stacked arrangement which is relevant to the mechanical properties of bone.

Citrate is abundant in bone, representing 1–5 weight percent (wt%) of the organic components with a density of approximately 1 molecule per 2 nm^2^, which implies that more than 15% of the apatite surface area available in bone is covered by citrate molecules [38]. Before the introduction of modern analytical techniques, the testing methods to evaluate bone tissue composition were entirely limited to classic wet-chemical analyses. At that time, the evidence of a higher amount of the citrate metabolic activity in bone as compared to other tissues, such as kidney or liver, was a significant breakthrough. These findings allowed hypothesising that bone tissue was endowed with the special capacity of producing and storing a high concentration of citric acid, and suggested the role of citrate in regulating the mineralisation process [39]. On the one hand, citrate may influence the amount of mineral deposition by complexing calcium-phosphate (CaP) and favouring its precipitation; on the other hand, if citrate exceeds the amount incorporated in the bone matrix, it could even reverse the mineralisation phase, thus functioning as a solubilising agent which recalls calcium ions. Hu et al. (2010) have proven that citrate is not a dissolved solubilising agent but is firmly bound to apatite as an integral part of the nanocrystal structure. This could be explained by the fact that the elevated amount of citrate in the organic fraction (5.5 wt%) provides more COO- groups than all the noncollagenous proteins in bone, and therefore, the chances of the binding between citrate and calcium of apatite are proportionally increased [40]. Simultaneously, Xie and Nancollas (2010) proposed a three-phase model to explain how citrate may influence the size, longitudinal growth and thickness of apatite nanocrystals [38]. The biomineralisation process initiates with the formation of the amorphous CaP phase starting from an oversaturated CaP solution [41] (Figure 3A). At the early stage, few citrate molecules can bind with the surface of small-size amorphous CaP clusters, but even so, they are sufficient to slow down particle aggregation (Figure 3B). In the next phase, the noncollagenous proteins released from bone cells promote the amorphous CaP cluster clumping, and apatite nucleation starts within these larger amorphous aggregates. Moreover, the presence of collagen fibrils promotes the self-assembly of the small amorphous CaP clusters and guides their direction on the collagen surface [42] (Figure 3C). At the final stage, the surface of mature apatite nanocrystals is fully covered by citrate, so that the increase in thickness is inhibited whilst the growth may continue in a longitudinal direction, thus explaining the plate-like morphology which apatite crystals exhibit in bone [38]. The final apatite structure has a unique geometry since it does not exceed 30–50 nm in length and maintains 2–6 nm thickness [43].

By using a combination of solid-state Nuclear Magnetic Resonance spectroscopy, powder X-ray diffraction, and the principles of electronic structure calculations, Davies et al. (2014) postulated that citrate anions could be incorporated in a hydrated layer of the CaP structure. This binding configuration favours the growth of the mineral crystals in a plate-like morphology and explains their propensity to form stacks [44,45] (Figure 3D). More recently, Delgado-Lopez et al. (2017) focused on the early mineralisation phase, taking into account the interaction between citrate and collagen [46]. By means of an in-depth characterisation based on X-ray scattering and imaging techniques, they found that collagen and citrate work synergically to favour the platy morphology, since both contributed to maintaining the transient amorphous phase. The amorphous CaP clusters are figured as spherulites or globules which gradually occupy the gap zones of the aligned collagen fibrils [43].

The results of the above-mentioned studies confirmed that citrate is an integral part of the apatite-collagen nanocomposite, and the degree of incorporation into bone mineral, as well as the spatial orientation in the mineral structure, play a key role in maintaining the biomechanic properties of bone, i.e., stability, strength, and resistance to fracture. In light of its structural role, citrate has been used to explain the changes in the bone microarchitecture typical of some diseases [47]. In addition, a series of citrate-based materials for orthopaedic applications have been developed to favour the osteoinductive and osteoconductive properties of scaffolds for bone tissue engineering [48,49], as well as to promote bone healing in other surgical procedures [50,51,52].

### 3.2. Citrate and Bone Cells

Once the presence of high concentrations of citrate in bone tissue was proven and the role of citrate in the mineralisation process clarified, the main goal of research in this field was to understand what the source of citrate in bone tissue was. Costello et al. (2012) demonstrated that murine osteoblasts were able to secrete citrate [53], thus laying the groundwork for additional studies which elucidated the metabolic process. Basically, the mechanisms were similar to those demonstrated in the prostatic cell, which is the other highly specialised cell capable of releasing high levels of citrate into the extracellular fluid. The elevated level of citrate production is due to the low activity of mitochondrial aconitase. This enzyme activity is suppressed by the zinc (Zn^2+^) that is accumulated by cells through the solute carrier 39A (SLC39A1), also known as “zinc importer protein 1” (ZIP1) [54,55,56].

Additional studies have confirmed that human osteoblasts were citrate-producing cells, that ZIP1 expression and intracellular zinc increased during the differentiation of the osteogenic precursors into bone-forming cells, that ZIP1 knockdown prevented the intracellular accumulation of citrate [5], and that ZIP1 expression was promoted by bone morphogenetic protein 2 during the mineralisation process [57]. In addition, the upregulation of the mitochondrial solute carrier 25A (SLC25A1), the citrate transport protein (CTP), was essential for the release of citrate from the mitochondria [56]. In order to define the gene expression patterns underlying the differentiation of mesenchymal stem cells (MSCs) into mature osteoblasts, a large-scale transcriptome analysis starting from the bone-marrow MSCs maintained in osteogenic medium up to deposition of mineral nodules was carried out [58]. While the role of ZIP1 was already described [5,56,59], the microarray analysis showed that solute carrier 13A (SLC39A13), or ZIP13, was upregulated throughout osteogenic differentiation and was also detectable during the mineralisation process [60].

**Figure 4:**
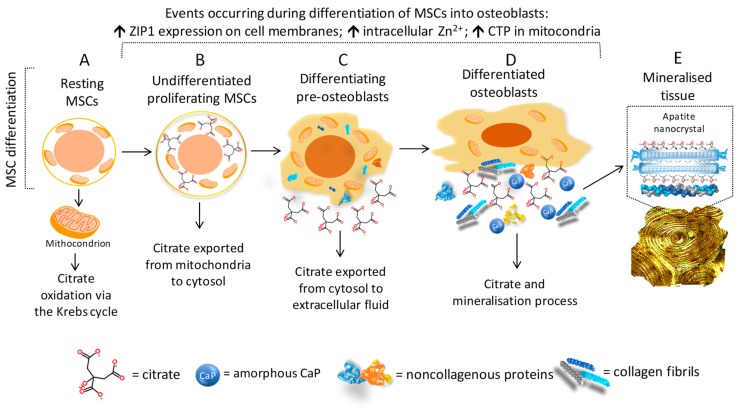
Citrate metabolism, osteoblast differentiation, and mineralisation process. The figure combines the concept of “osteoblast citration” with the main steps of the differentiation of mesenchymal stem cells (MSCs) into bone-forming cells (osteoblasts) [5,53,56]. (**A**) Resting MSCs are quiescent, nonproliferating cells which exhibit the typical mitochondrial metabolism with the oxidation of citrate via the Krebs cycle. (**B**) In the presence of proper stimuli, the undifferentiated MSCs are committed to osteogenic differentiation and, at the early phase, high proliferation is required. To accomplish this goal, the following events are necessary: (1) the upregulation of ZIP1 which promotes the zinc intake, (2) the accumulation of mitochondrial citrate due to the zinc-dependent inhibition of the mitochondrial aconitase, (3) the exportation of citrate into cytosol by means of the “citrate transport protein” (CTP/SLC25A1), (4) the use of cytosol citrate for the lipogenesis process which is essential for cell duplication. (**C**,**D**) The citrate exportation from cytosol to extracellular fluid starts during cell differentiation, and it is simultaneous for the synthesis and the release of amorphous CaP, collagen, and noncollagenous proteins. (**E**) The “osteoblast citration” is completed when the mineralised matrix is assembled. The role of citrate in growing the apatite nanocrystals and driving the mineralisation process is explained in Figure 3.

As shown in Figure 4A–E, changes in the citrate metabolism occur during the whole process of differentiation of the MSCs into mature osteoblasts. After the osteogenic commitment of resting MSCs, a highly proliferative phase is expected, and the exportation of citrate into cytosol provides the acetylCoA for the synthesis of the new lipids required for the assembly of the new plasma membranes during cell duplication. Mitochondrial citrate seems to be the preferential source of acetylCoA, since the alternative sources pose some limitations. In fact, extracellular citrate could be used for lipogenesis; however, cellular uptake is subordinated to the expression of the specific transporter, i.e., “sodium-dependent citrate transporter” (NaCT; SLC13A5) [61]. AcetyilCoA could also be obtained from plasma acetate passing through the cell membrane by means of the monocarboxylate transporter (MCT/SLC16A). In order to use this source, the upregulation of acetylCoA synthetase is required, but it is not highly expressed in mammalian cells [56]. Moreover, aconitase inhibition and the lack of mitochondrial citrate affect the energy supply via the Krebs cycle, so that the bioenergetic demand for proliferating/differentiating cells has to be satisfied by alternative sources. For instance, cytosol malate may enter the mitochondrion by CTP in exchange for citrate and may be converted into oxalacetic acid, which in turn may originate new citrate [56].

On the basis of recent studies, citrate released during the bone resorption phase could be considered a matrix-derived signal which contributes to the overall process of bone remodelling within the “basic multicellular unit” [62]. In this regard, Ma et al. have demonstrated that extracellular citrate played a pivotal role regarding the osteogenic differentiation of MSCs [63]. This “metabonegenic” regulation started with citrate uptake through the sodium-citrate transporter SLC13A5 followed by the activation of energy-producing pathways leading to an elevated cell energy status, which in turn fueled the high metabolic demands of MSCs differentiating into osteoblasts. In vitro experiments have shown that the timing and dosage of the citrate supply were critical factors. In fact, the effects were dose-dependent and more evident at the early stages of osteogenic differentiation, with higher proliferation and increased expression of bone-related genes, i.e., alkaline phosphatase and alpha 1 chain of type I collagen [63]. Identical effects have also been observed in a microenvironment hostile to bone cells, for instance using culture conditions which simulated low-grade acidosis [64]. In fact, citrate supplementation opposed the detrimental effects resulting from extracellular acidosis, which inhibited the synthesis of collagen and non-collagen proteins, the activity of alkaline phosphatase, and the formation of mineral nodules [65].

Taken together, studies regarding the role of citrate in the nanocrystal structure and those showing that osteoblasts were the specialised citrate-producing cells in bone have led to a new concept of bone formation related to “citration”. Briefly, Costello et al. (2012) stated that….”when considering the mineralisation role of osteoblasts in bone formation, it now becomes evident that ’citration’ must be included in the process. Mineralisation without ‘citration‘ will not result in the formation of normal bone, i.e., bone that exhibits its important properties, such as stability, strength, and resistance to fracture” [53].

## 4. Citrate Pathophysiology and Bone Diseases

The role of citrate in mineralised tissues poses several questions regarding the consequences of a low bioavailability at the systemic level. For the most part, published data linking citrate alteration with bone metabolism refer to renal diseases, acid-base imbalance or also physiological conditions such as menopause, but there are also inheritable genetic defects which affect the TCA cycle in mitochondria or the citrate transport. In the following paragraphs, the medical conditions in which the association between citrate and bone health status has been implied are discussed.

### 4.1. Bone Health Status and Alterations of Citrate Homeostasis in Kidney Diseases

With the progressive aging of the population, epidemiological studies have shown a higher rate of elderly-related illnesses, including the impairment of bone quality leading to osteoporosis and decreased renal function with chronic kidney disease (CKD), which in turn may influence bone health status [66,67,68]. The decrease in renal function may be mild, moderate or severe on the basis of estimated-glomerular filtration rate (GFR) equations and is associated with the simultaneous impairment of mineral homeostasis, including serum and tissue concentrations of phosphorus and calcium, circulating levels of calciotropic hormones (PTH, 25-hydroxyvitamin D, 1,25-dihydroxyvitamin D), fibroblast growth factor-23, and growth hormone. The modifications of mineral homeostasis may promote a loss of bone mass and an increase in bone fragility [69]. As observed by Malmgren et al. (2015), approximately 95% of women over 75 years of age showed a mild-moderate decrease in renal function (CKD stages 2–3) which may have had a harmful effect on bone health [70]. In a 10-year longitudinal study, the authors evaluated the long-term influence of impaired renal function on bone mineral density (BMD) [71]. They analysed 1044 Caucasian women from the “Osteoporosis Prospective Risk Assessment” (OPRA) cohort and found that renal function was positively correlated with femoral neck BMD in elderly women, although the association attenuated as aging progressed. Women with poor renal function had a higher annual rate of bone loss over 5 years compared to those with normal function, and markers of mineral homeostasis were more frequently altered.

High-throughput “omics” approaches, including metabolomics, have been proposed to identify new biomarkers which could help the management of CKD patients, and TCA cycle-metabolites are emerging as potential candidates [72]. A significantly reduced urinary excretion of citrate (–68%) has been observed in non-diabetic patients with CKD as compared to subjects with normal renal function. The renal expression of genes regulating the TCA cycle was decreased in subjects who had impaired renal function, thus suggesting that mitochondrial dysfunction could be involved in the pathogenesis of CKD [73]. Moreover, GFR positively correlated with citrate excretion, and kidney stone formers with CKD had significantly lower urinary citrate excretion than subjects with kidney stone disease and normal renal function [74].

To the authors’ knowledge, the link between CKD, urinary citrate and bone health status has still not been elucidated but, taking into account the information emerging from the previous paragraphs, it is reasonable to assume that the link exists. Some indications have derived from the data regarding kidney stone disease, which is the paradigmatic expression of a relationship between citrate alterations, BMD decrease and fracture risk that has been investigated since the 1970s [75]. In fact, several studies have shown that osteoporotic fractures occurred more frequently in patients with kidney stones than in the general population [76,77,78,79].

The connection between kidney stones and bone metabolism is related to several factors. Briefly, kidney stones form when urine becomes supersaturated with respect to its specific components. Since 80% of kidney stones are composed of calcium-oxalate (CaOx) or CaP, the regulation of calcium excretion plays a pivotal role in the etiopathogenesis of nephrolithiasis [80]. As urinary citrate is able to bind calcium and prevent the growth and agglomeration of CaOx and CaP crystals, the close relationship between low citrate excretion and kidney stone formation has been fully established [11]. The incidence of hypocitraturia varies from 20% to 60% in people who have a propensity to form stones, either as a single abnormality or in conjunction with other metabolic disorders [16]. Hypercalciuria may occur either when filtered calcium is abnormally increased or when its reabsorption is abnormally decreased. The former may be associated with enhanced bone resorption which raises calcium bioavailability at the systemic level, while the latter may be the consequence of decreased renal function as occurs in CKD. Theoretically, reduced GFR in CKD should lead to decreased urinary calcium concentration, but the consequences of defective tubular reabsorption are more relevant and are responsible for the supersaturation of calcium salts. In addition, in the distal nephron, calcium reabsorption is a PTH-dependent process, PTH being the hormone capable of stimulating the resorption of the bone matrix in response to low, systemic calcium availability [81]. Therefore, as the decrease in the renal function progresses, PTH levels and bone loss gradually increase, thus explaining why kidney stones are a significant predictor of osteoporotic fracture in patients with CKD [82]. Moreover, when nephrolithiasis occurs, patients are frequently advised to reduce calcium intake, thus favouring a negative calcium balance which is an additional risk factor promoting a decrease in BMD [11].

Recent findings have demonstrated that lithogenic risk factors, including hypocitraturia, are also detectable in patients without kidney stones who exhibit osteoporosis or osteopenia, thus leading to the hypothesis that the evaluation of lithogenic risk could have significant implications for monitoring bone health status [34,83].

### 4.2. Postmenopausal Osteopenia and “Net Citrate Loss”

Estrogen deficiency and aging are the main factors responsible for the depletion of bone mass [84], but they are also associated with changes in urine composition which are similar to those of subjects having an increased risk of kidney stones [11]. The circulating citrate levels and the citrate content in bone are markedly reduced in animals with age-related or ovariectomy-induced bone loss [85]. A low citrate excretion, less severe than true hypocitraturia fixed at less than 320 mg per day, has been described in postmenopausal women [11,33] and in subjects with a low bone mass [34,83]. Nurses’ Health Study II considered an ongoing cohort of 108,639 participants from whom information on menopause and kidney stones was obtained. In general, postmenopausal status was associated with lower BMD and a higher incidence of kidney stones in this cohort. Moreover, small but significant differences in urine composition were found in 658 participants who had pre- and postmenopausal 24-h urine analyses, including a lower citrate excretion [86].

The postmenopausal decline in estrogen concentration influences the activation rate of basic multicellular units composed of bone-resorbing osteoclasts and bone-forming osteoblasts. However, according to Drake et al., resorption increased by 90% while formation increased by only 45% [87] and the final result was a “net bone loss”. This imbalanced bone remodelling depends on the effects that the lack of estrogen has on bone cells. On the one hand, the activity of the receptor activator of the nuclear factor-κ B ligand (RANKL) is promoted, a key factor in osteoclast differentiation; on the other hand, the osteogenic precursors are destined to differentiate into adipocytes, and the survival of mature osteoblasts is suppressed [88]. The result is the reduction of mature osteoblasts, and since they are the cells capable of synthesising citrate [5], the consequence is lower citrate production which impairs the quality and the stability of the bone microarchitecture [38,40]. Moreover, osteoclast differentiation and bone resorption are energy-demanding processes, and the citrate which is synthesised cannot be accumulated because it is essentially utilised through the citric acid cycle [89,90]. Similarly, the MSC differentiation towards adipocytes requires more citrate as a source of cytosolic acetylCoA for lipid biosynthesis [85]. In conclusion, according to Granchi et al., estrogen deficiency leads to a “net citrate loss” which could explain the diminished citrate excretion observed in postmenopausal women [91].

### 4.3. Genetic Variations Influencing Citrate Homeostasis and Skeletal Development

The “Online Mendelian Inheritance in Man^®”^ database (OMIM^®^) is a comprehensive repository of information on the relationship between genetic variation and phenotypic expression [92]. The annotations connecting citrate homeostasis with skeletal defects are listed in Table 2, and many of these concern Slc proteins, which are a family of solute transporters through the membranes.

Mutations of the Cl2/HCO3·2 exchanger AE1, encoded by SLC4A1 which is expressed in red blood cells and in type A intercalated cells of the renal collecting tubule, may be responsible for distal renal tubular acidosis (dRTA), with or without haemolytic anemia. The corresponding phenotype displays defective urine acidification, nephrocalcinosis, nephrolithiasis, hypercalciuria, and hypocitraturia [93]. The clinical phenotype in patients with inherited dRTA is characterised by stunted growth with bone abnormalities in children, as well as nephrocalcinosis and nephrolithiasis which develop as the consequence of hypercalciuria, hypocitraturia, and relatively alkaline urine.

The same cytogenetic location of SLC4A1 (17q21.31) is involved in Glycogen storage disease Ia which is caused by a deficiency in glucose-6-phosphatase activity that catalyses the synthesis of glucose from glucose-6-phosphate. This enzymopathy results in a failure to maintain normal glucose control with glycogen accumulation in the liver, kidney, and intestine. Low citrate excretion and hypercalciuria have been described by Weinstein et al. (2001), and the combination of these metabolic alterations correlated with the onset of nephrocalcinosis and nephrolithiasis [94]. Furthermore, there is increasing evidence that poor metabolic control, including chronic acidosis (lactic), low muscle mass and delayed puberty, may negatively affect BMD in half of the patients [95].

NaCT is the sodium-coupled tricarboxylate transporter predominantly expressed in the liver, at several-fold lower levels in the testis and the brain, and at weak levels in the kidney and the heart. The association between the mutations of the SLC13a5 gene on chromosome 17p13 and early infantile epileptic encephalopathy-25 with amelogenesis imperfecta has been clearly recognised [96]. More recently, Irizarry et al. have shown that SLC13a5 deficiency led to decreased BMD and impaired bone formation in homozygote (Slc13a5-/-) and heterozygote (Slc13a5+/-) mice [97]. As shown by Diaz et al. (2017), the epigenetic modulation of SLC13a5 gene may also influence skeletal development, since DNA hypermethylation and low gene expression have been found in the placenta and cord blood of infants born small-for-gestational-age and correlated with low height and weight at birth, low BMD, and low mineral content [98].

Bartter syndrome refers to a group of autosomal recessive disorders characterised by impaired salt reabsorption in the thick ascending limb of the loop of Henle with pronounced salt wasting, e.g., potassium and calcium, and hypokalemic metabolic alkalosis [99]. The antenatal variant or Bartter syndrome type I is caused by a homozygous or compound heterozygous mutation in the sodium-potassium-chloride cotransporter-2 gene [100]. The affected infants develop marked hypercalciuria and, as a secondary consequence, nephrocalcinosis and osteopenia [101]. To the best of the authors’ knowledge, low citrate excretion in patients affected by Bartter syndrome has not been described, but citrate potassium administration is able to correct biochemical alterations [102,103].

Familial hypomagnesemia with hypercalciuria and nephrocalcinosis is an autosomal-recessive renal tubular disorder caused by mutations in claudin-16 and claudin-19, which are members of the transmembrane family proteins regulating calcium and magnesium reabsorption in the kidney [104]. Thorleifsson et al. have also identified claudin-14 as a major risk gene of hypercalciuric nephrolithiasis associated with a decrease in BMD [105]. Patients can develop hypomagnesaemia, hypercalciuria, and nephrocalcinosis, and their clinical course is often complicated by the progressive loss of kidney function. Other biochemical anomalies consist of elevated serum PTH levels before the onset of CKD, incomplete distal tubular acidosis, hyperuricemia and hypocitraturia [106]. Additional symptoms may be recurrent urinary tract infections, nephrolithiasis, polyuria, polydipsia and/or failure to thrive [106]. Amelogenesis imperfecta has also been described in some patients [107].

The human gene SLC13A2 encodes the sodium-dicarboxylate cotransporter (NaDC1) which is highly expressed in the brush-border membranes of the renal proximal tubule and intestinal cells, and reabsorbs Krebs cycle intermediates, i.e., succinate and citrate [108]. Although to date no distinctive phenotype has been linked with SLC13A2 variation in the OMIM database, Okamoto et al. (2006) have hypothesised that NaDC1 alterations could play a role in the development of kidney stones by affecting the citrate concentration in the urine [109]. The functional properties and protein expression of eight coding region variants of NaDC1 have recently been characterised; the majority of them appeared to decrease transport activity and were predicted to result in decreased citrate absorption in the intestine and kidney [110]. Even if not investigated, it is reasonable to assume that effects on bone mass may occur since these conditions influence citrate metabolism and predispose to renal stone formation as well.

The mitochondrial CTP, coded by the SLC25A1 gene located on chromosome 22q11.21, is embedded in the inner membrane and determines the efflux of tricarboxylic citrate from the mitochondria to cytosol in exchange for dicarboxylic malate [111]. The high citrate concentration into cytoplasm modulates the lipid synthesis and affects glycolysis by inhibiting phosphofructokinase-1 [112]. Genetic variations of SLC25A1 mainly lead to inheritable diseases featured by alterations of the central nervous system (combined D-2- and L-2-hydroxyglutaric aciduria; OMIM ID: 615182) and skeletal muscle (congenital myasthenic syndrome-23; OMIM ID: 618197) while the presence of bone defects is less relevant. However, SLC25A1 impairment also occurs in the 22q11.2 deletion syndrome which is characterised by congenital absence of the thymus and parathyroid glands as well as cardiac, renal and eye anomalies, developmental delay, and also skeletal defects. As additional evidence of a relationship between citrate transport and bone pathophysiology, it has been shown that SLC25A1 knockout in mice causes a notable decrease in the number of osteoblasts and the amount of osteoid [113].

As mentioned above, zinc plays a crucial role in regulating the extracellular bioavailability of citrate in the formation of new mineralised matrix and, therefore, gene defects involving a zinc transporter may be involved in alterations of the citrate metabolism and bone diseases. Of these, the solute carrier 39A family (SLC39A or ZIP) controls the influx of zinc into the cytoplasm [56]. In a previous study, the authors found that SLC39A13 (ZIP13) is upregulated throughout osteogenic differentiation, and no changes were recorded during the mineralisation process [58]. SLC39A13 gene defects have been associated with low bone mass in knockout mice [56] and spondylodysplastic Ehlers-Danlos syndrome, type 3 (Phenotype MIM number 612350).

## 5. Medical Management of Patients with Metabolic Bone Diseases Associated with Citrate Alterations

### 5.1. Clinical Work-Up

At present, the guidelines dealing with the clinical management of metabolic bone diseases do not highlight the role of citrate in maintaining bone integrity. Nevertheless, based on research findings, the causes of hypocitraturia should be considered in carrying out a complete evaluation of patients who present BMD alterations; vice versa, the accurate monitoring of BMD is advisable in subjects who have reduced urinary citrate excretion.

Regarding laboratory investigations, citrate homeostasis should be evaluated together with all factors which influence mineral metabolism. Although citrate excretion must be measured over a 24-h period and referred to the 24 h-urine volume, the detection of citrate levels in fasting-morning urine (expressed as creatinine ratios) may be an addional element to complete the metabolic profile. Urine pH can be a valid and simple indicator of acid-base balance. Laboratory testings for evaluation of mineral metabolism have to be carried out on plasma and urine by considering renal function, calcium-phosphorus balance, other electrolytes (in particular, potassium and magnesium), and calciotropic hormones. In addition, bone turnover markers (BTMs), including bone-resorption and bone formation indicators, are considered a useful and inexpensive tool for evaluating turnover rate (high or low) and response to any treatment [114].

Quantitative assessment of BMD is mandatory for evaluating bone health status and for calculating fracture risk [115]. Dual-energy X-ray absorptiometry (DXA), the T score (the number of standard deviations above or below the mean for a healthy 30-year-old adult of the same sex and ethnicity), or the Z-score (the number of standard deviations above or below the mean for the patients of the same age, sex and ethnicity) are employed for measuring areal density (g·cm^−2^) at any skeletal site. The assesment of bone quality may be additionally explored by evaluating the trabecular bone score which reflects bone microarchitecture [116]. “High-resolution peripheral quantitative computed-tomography” allows an accurate assessment of bone strength; however, due to its elevated cost, the diffusion of this technology is still limited.

Even though noninvasive techniques are preferred, bone biopsy remains a valid tool for assessing the tissue quality in metabolic bone diseases, and it is the gold standard for estimating bone impairment in kidney disease and for guiding the clinician in deciding proper treatment [117]. In fact, according to the Kidney Disease Improving Global Outcomes (KDIGO) guidelines, the term renal osteodystrophy may be used exclusively to define the histological alterations in bone morphology associated with CKD, which can be additionally assessed using histomorphometry. Instead, without histological confirmation, the clinical syndrome which develops as a systemic disorder of the mineral and the bone metabolisms is generally called “CKD-Mineral and Bone Disorder” [69,118]. However, bone biopsies in a routine work-up present some drawbacks, which are foremost the availability limited to specialised centres, discomfort for the patients, and the length of time required to process and analyse bone tissue.

Whenever instrumental and laboratory investigations show a significant bone loss, pharmacological treatment has to be planned according to the indications of the current guidelines, eventually adding specific drugs in case of secondary osteoporosis. Moreover, all recommendations related to lifestyle and dietary modifications should be explained to the patient [115]. In the presence of low urine citrate excretion, citrate-based supplements could be recommended to prevent progressive damage to bone and a progressive reduction in BMD.

### 5.2. Dietary Modification

Since hypocitraturia may depend on food habits (Table 1), dietary modifications should be considered as a first level intervention for the medical management of citrate deficiencies. Diet is aimed at correcting the excessive acid load and, as a consequence, the negative effects that acidosis has on bone metabolism [119]. The acid-ash hypothesis emphasises the role of the skeleton in maintaining the acid-base balance since the hydroxyapatite of the mineral matrix is a reservoir of alkali groups which may be released to neutralise proton excess [120]. Acute and chronic acid loading show distinct effects, with acute acidity first eroding the bone surface to release sodium, potassium and bicarbonate into the circulation, while chronic acidity leads to the release of calcium and phosphate [121]. Moreover, acidosis directly influences the activity of bone cells within the bone remodelling unit by promoting osteoclast-mediated resorption and inhibiting bone formation by osteoblasts [65]. Hypocitraturia is a response to the elevated acid load occurring in metabolic acidosis, since there is a notable increase in citrate reabsorption in the renal proximal tubule when the tubular pH decreases [16]. In this regard, there is consensus in considering citraturia as a biomarker for monitoring diet and the metabolism-dependent systemic acid-base status, even in subjects without overt metabolic acidosis [33,34,35].

Under physiological conditions, the acid-base balance is strictly controlled by net endogenous acid production related to acid and alkali dietary intake, and incomplete metabolism of organic acids. By using proper methods, such as “net endogenous acid production” (NEAP) [122] and “potential renal acid load” (PRAL) [123], it is possible to determine the production of acids and characterise foods according to their ability to release acids and bases into the bloodstream. The prolonged and excessive consumption of acid precursor foods leads to chronic low-grade metabolic acidosis which reduces the excretion of citrate and predisposes to diseases [124], including alteration of bone health status, especially in older subjects with diminished renal function [125]. Some authors have discarded the acid-ash hypothesis, claiming that the increase in the diet acid load did not promote skeletal bone mineral loss or osteoporosis [126], and the main role in regulating acid-base homeostasis should be attributed to the kidney [127]. However, the above studies did not consider the role played by citrate in preserving the mineralised matrix and it would be interesting to know whether the relationship between acid dietary intake and alterations in the bone metabolism varied according to low or normal urine citrate excretion. As supporting evidence for the link between citrate and bone health, previous studies have demonstrated a positive correlation between citrate excretion and radius densitometric values in pre- and postmenopause, as well as a significant relationship between citraturia and the prevalence of vertebral fracture in postmenopausal women [11].

The general dietary approach to counteract elevated acid load is to limit the intake of foods with a high acidifying potential, i.e., meat (beef, pork, poultry), fish and seafood, eggs, beans and oilseeds, in favour of foods which contribute the most to the release of bases, i.e., fruits and vegetables [128]. However, dietary modifications cannot disregard the medical history of the patients, and the best nutritional approach should be evaluated on a case-by-case basis. For instance, in the elderly, inadequate protein intake could be a greater problem for bone health than protein excess [129], and the intake of high amounts of fruits and vegetables could be contraindicated in patients with CKD due to their high potassium content [130].

The intake of foods with a high citrate content may be a valid approach to compensate for the high demand due to acidosis or other causes of hypocitraturia [24,124]. The daily citrate intake is approximately 4 grams, and almost all citrate introduced by exogenous sources is absorbed into the gastrointestinal tract, arrives in the liver and is metabolised to bicarbonate [11].

Prezioso et al. (2015) examined the relationship between a diet rich in vegetables and urinary citrate excretion [24]. Fruits and vegetables (except for those with high oxalate content) favour citrate excretion; consequently, they decrease urinary saturation for CaOx and CaP, thus having a protective effect on the formation of kidney stones [24]. In general, fruit intake is lower in hypocitraturic than in normocitraturic subjects [131].

In order to provide dietary recommendations aimed at correcting hypocitraturia, Haleblian et al. (2008) carried out an exhaustive analysis of citrate concentrations in citrus juices, noncitrus juices, and commercially available beverages. The highest concentration was found in grapefruit juice (35% more than in lemon juice), and a glass corresponded approximately to a 40 mEq tablet of potassium citrate. In general, commercial beverages had lower amounts of citrate [7].

Several authors have studied the possible influence of the consumption of fruit juices (both citrus and noncitrus) on urinary citrate excretion. Orange juice increased the excretion of urinary oxalate, and therefore, its consumption could result in the biochemical modification of stone risk factors [132]. It should also be noted that grapefruit juice significantly increased urinary oxalate levels, but it was not associated with an increased lithogenic risk probably due to the protective effect of the high citrate content [133]. Regarding noncitrus juices, cranberry juice had a controversial effect on urine citrate (no effect or an increase of 31%), but resulted in a significantly increased concentration of urinary calcium and oxalate. In addition, diluted blackcurrant juice and melon had a positive effect in increasing citraturia [24]. In a recent meta-analysis, Pachaly et al. aimed at systematically investigating the effects of dietary measures on urinary citrate and nephrolithiasis [124]. They searched for randomised controlled and crossover studies which evaluated urine citrate excretion after the intake of citrus-based beverages, including fruit juices and soft drinks, calcium/magnesium rich mineral water, a high-fibre diet, a low-animal-protein diet, and plant extracts. The authors identified thirteen studies involving 358 participants, the majority of whom were stone formers. Summarised estimates showed a significant increase in citraturia levels only in subjects who consumed fruit juice and other beverages while the other dietary modifications did not determine significant changes [7,8].

Clinical trials aimed at evaluating whether an increase in dietary citrate preserved the bone health status are lacking. In a clinical trial, postmenopausal women were randomised into four groups, i.e., a diet (additional daily portion of 300 g of self-selected fruit and vegetables), two doses of potassium citrate (12.5 and 55.5 mEq/day) and a placebo (control group). The participants were followed for two years, and the effects on bone turnover were determined by measuring BTMs and BMD. The authors concluded that neither potassium citrate nor fruit and vegetables influenced bone turnover or prevented BMD loss over 2 years in healthy postmenopausal women (Table 3) [134].

In summary, natural sources of dietary citrate should be considered as a first option for preventing kidney stone recurrence as an alternative to medical treatment [24]. From a theoretical point of view, published data have suggested that dietary modifications could also be effective in preserving bone health. However, at present, there is insufficient evidence supporting the use of natural sources of citrate as the sole treatment for preventing bone loss.

### 5.3. Citrate-Based Supplements

Nephrolithiasis was the first clinical condition in which oral citrate supplementation showed therapeutic efficacy, particularly in lowering high stone recurrence rate which is more prevalent in people with low urinary citrate levels [11]. The rationale for using citrate salts in kidney stone disease was explained in previous paragraphs and was based on four main issues: (1) citrate salts are rapidly absorbed through the intestine and equally rapidly filtered in the urine; (2) citrate forms calcium citrate complexes, which in turn increase solubility and decrease the amount of free calcium in urine; (3) citrate acts as an inhibitor of CaOx and CaP crystal growth and aggregation, and (4) in the intestine, the complexation between calcium and citrate reduces enteric absorption of calcium, and therefore renal excretion. A recent systematic review has demonstrated that citrate-based therapy reduced recurrent calcium urinary stone formation compared to controls (placebo, usual care) [135]; however, evidence was limited in children [136].

There have also been interventional clinical trials concerning the effect of citrate supplements in preserving bone health status. As stated for dietary modifications, the rationale of the published trials was based on the assumption that citrate-based supplements may be useful as alkalising agents which neutralise the effects of an excessive acid load [16,65,119,120,121]. Other than diets rich in salt and animal protein [128], several conditions may induce low-grade acidosis, and the majority of them are age-related, such as menopause [33], subclinical inflammatory status [137], and decreased renal function [68]. On the basis of this assumption, Lambert et al. (2015) carried out a meta-analysis aimed at determining whether alkaline potassium salts, including potassium citrate and potassium bicarbonate, had some effect on calcium metabolism and bone health [138]. The seven eligible studies dealing with potassium citrate supplementation did not include subjects with nephrolithiasis and/or other relevant comorbidities in order to avoid confounding factors potentially leading to overestimation or underestimation of the intervention. Citrate salts significantly reduced calcium and acid excretion similarly to potassium bicarbonate, but they seemed to be more effective in preventing collagen resorption. However, insufficient data were available regarding changes in BMD, since it was investigated in only two studies. The authors found major differences in terms of study design, inclusion/exclusion criteria, doses, timing of supplement administration and outcome measures; this heterogeneity represented a notable limitation for translation into a clinical setting.

In the current review, the interventional clinical trials which were primarily aimed at evaluating the effect of citrate supplements on mineral metabolism and bone turnover were reviewed. Sixteen eligible studies were identified which (1) recruited more than 10 subjects, (2) excluded nephrolithiasis and other significant comorbidities and (3) reported the results related to bone health status, including BMD and/or BTMs. Data regarding study design, population, intervention, follow-up, additional supplementation or controlled dietary intake, as well as a summary of results and conclusions, are shown in Table 3.

The authors investigated the effect of potassium citrate (one clinical trial, seven randomised clinical trials (RCTs)) [92,134,139,140,141,142,143,144], calcium citrate (four RCTs and two crossover studies) [145,146,147,148,149,150], and two studies compared the above-mentioned treatments (one RCT, one crossover study) [139,140]. Regarding the intervention under investigation, doses, timing of administration, and follow-up varied greatly among the trials and, in thirteen studies, the co-administration of additional supplements was planned, i.e., calcium carbonate and/or vitamin D3. The majority of the studies included postmenopausal women (*n* = 11) while, in one study, the participants were of childbearing age. The other trials were designed to recruit healthy individuals, i.e., males and females at least 55 years of age. The dietary intake of calcium and/or protein and/or salts was controlled in ten studies; in five studies, the subject consumed a free nonvegetarian diet; in one study, the management of food habits was not described.

Overall, the studies selected provided more than 1000 experimental cases and more than 900 controls. As Lambert et al. reported previously, very high heterogeneity among the studies was observed, and therefore the meta-analysis lost its inferential value while the descriptive overview was more informative. By examining the conclusions reported by various authors, two recurring key points emerged: (1) an adequate calcium intake was essential in preventing bone loss in elderly subjects and in postmenopausal women, and calcium citrate seemed to be more effective than calcium carbonate [145,146,147,148,149,150] and (2) potassium citrate prevented the increased bone resorption caused by menopause, chronic acidemia and high salt intake [139,140,141,142,143,144].

Additional findings regarding potassiun citrate can be summarised as follows: effectiveness may be enhanced by combined treatment with calcium citrate [139]; positive effects have also been observed in young women and in the absence of an excessive acid load [140]. Moreover, supplementation with alkalising potassium citrate improved the beneficial effects of calcium and vitamin D only in osteopenic postmenopausal women who exhibited the target conditions, namely low potassium and/or citrate excretion and/or low urine pH [91]. Finally, one study questioned the beneficial effects of potassium citrate since, at 24 months, no significant modifications in BTMs and BMD were recorded [134]. It should be noted that compliance may decrease by 25% over time, mainly due to gastrointestinal side effects (approximately 10%) and costs [24].

Despite the encouraging results, a consensus statement regarding the use of citrate supplementation for the management of metabolic bone diseases is still lacking since the heterogeneity of the current evidence is a major limitation for identifying the best practice. Interestingly, the identification of subjects who better respond to exogenous citrate supplementation could be the correct way to maximise the beneficial effect of the treatment.

## 6. Conclusions

Since citrate is a ubiquitous metabolite with a multidimensional role in the human organism, it is not surprising that it is involved in the pathophysiology of tissues, organs and systems. In fact, recent data in the literature have highlighted the relationship between citrate defects and various medical conditions with considerable financial and social impact, e.g., cancer [153], dyslipidemia [154], vascular calcifications [155], and visual disabilities [156].

Overall, this review has highlighted the main functions of citrate, and, more specifically, focused on the role of citrate in the pathophysiology and medical management of metabolic bone diseases, thus identifying some key points which are summarised in Table 4.

Although interest in the role of citrate is spreading, a consensus statement regarding the use of citrate supplementation for the management of metabolic bone diseases is still lacking, since the heterogeneity of the current evidence is a major limitation in identifying the best practice. Even when the study design is well planned, there are some reasons which may explain the difficulty in obtaining unique and compelling results from clinical trials.

First, dietary supplementation cannot be regarded as a pharmacological treatment, and therefore the effects on measurable clinical outcomes are expected to be less prominent. Hence, baseline differences between participants which are beyond the investigator’s control become selection biases that attenuate the strength of randomisation and interfere with the interpretation of the results. On the other hand, current scientific knowledge provides a sufficient background, leading the clinician and the researcher to further investigate the beneficial effects of citrate-based treatment in depth, as proven by multiple studies which have dealt with this matter.

At present, it can be argued that the use of citrate supplementation should be considered in the medical management of bone diseases, but it is also reasonable to assume that the effects could be maximised in a personalised approach in which the scientific knowledge and the clinical judgement of practitioners are essential in identifying patients who could reap real benefits.

## Figures and Tables

**Table 1:** Causes of low citrate excretion.

Cause	Annotation
Acid-base status [16,23]	Acidosis increases citrate utilization by the mitochondria in the tricarboxylic acid cycle (TCA cycle), thus decreasing intra- and extracellular availability. As a consequence, citrate reabsorption is enhanced and urine excretion is reduced. On the contrary, alkalosis increases citrate elimination.
Hypokalemia [16,23]	Low potassium levels cause intracellular acidosis (see above).
Diet [24,25]	Low intake of high-citrate content food (fruit/vegetables). A diet rich in animal proteins contains sulfate and phosphate moieties which are not metabolised and are excreted as acids which decrease urinary pH and citrate excretion. High sodium intake, ketosis-promoting diet, and starvation.
Distal renal tubular acidosis (dRTA) [26]	Complete form (hyperchloremic metabolic acidosis, hypokalemia, elevated urine pH). Incomplete form (normal serum electrolytes, inability to acidify urine following an ammonium chloride load).
Chronic diarrheal syndrome [16,23]	The fluid loss and intestinal alkali wasting alter the acid-base status, with low urinary pH and citrate retention.
Medications [16,23,27,28]	Thiazide diuretics induce hypokalemia with resultant intracellular acidosis. Acetazolamide (carbonic anhydrase inhibitor) produces changes in urine composition which are similar to those found in dRTA. Angiotensin-converting enzyme inhibitors cause a reduction in urinary citrate by increasing the adenosine triphosphate (ATP) citrate lyase activity. Topiramate (carbonic anhydrase inhibitor) exerts a dose-dependent effect on the renal excretion of citrate.
Strenuous physical exercise [23]	It causes lactic acidosis, producing hypocitraturia.
Hyperuricosuria [23]	With normouricemia, generally caused by dietary excess of purines (animal proteins). With hyperuricemia (gouty diathesis), the urinary pH is typically low, with increased citrate reabsorption.
Active urinary tract infection [23]	Bacteria which degrade citrate lower the urinary citrate concentration.
Chronic kidney disease (CKD) [29]	The decrease in the glomerular filtration rate causes a stepwise reduction in the amount of filtered citrate. Overt hypocitraturia is usually observed in advanced stages of CKD.
Primary hyperaldosteronism [30]	Hypocitraturia (and hypercalciuria) occurs via Na-dependent volume expansion and chronic hypokalemia.
Menopause [31,32,33,34]	Estrogen deficiency induces metabolic alteration related to the lowering of estrogen-induced signaling onto the mitochondria which promotes glycolysis and glycolytic-coupled TCA cycle function. Hormone replacement therapy restores the citrate level which is decreased in postmenopausal women.
Genetic defects [16]	All inheritable diseases, gene defects, and polymorphisms associated with the above mentioned conditions (additional details in Table 2).

**Table 2:** Genes involved in the regulation of citrate homeostasis with a genotype/phenotype relationship regarding skeletal development and/or bone metabolism (retrieved from the OMIM® database, last access 25 May 2019).

Gene/Locus Name	Gene/Locus	Cytogenetic Location	MIM Number: Phenotype	Inheritance
Solute carrier family 4, anion exchanger, member 1 (erythrocyte membrane protein band 3, Diego blood group)	SLC4A1, AE1, EPB3, SPH4, SAO, CHC	17q21.31	179800: Distal renal tubular acidosis	Autosomal dominant
Solute carrier family 4, anion exchanger, member 1 (erythrocyte membrane protein band 3, Diego blood group)	SLC4A1, AE1, EPB3, SPH4, SAO, CHC	17q21.31	611590: Distal renal tubular acidosis	Autosomal recessive
Glucose-6-phosphatase, catalytic	G6PC, G6PT	17q21.31	232200: Glycogen storage disease Ia	Autosomal recessive
Solute carrier family 13 (sodium-dependent citrate transporter), member 5	SLC13A5, NACT, INDY	17p13.1	615905: Early infantile, epileptic encephalopathy, 25	Autosomal recessive
Solute carrier family 12 (sodium/potassium/chloride transporters), member 1	SLC12A1, NKCC2	15q21.1	60167: Bartter syndrome, type 1	Autosomal recessive
Claudin 16 (paracellin 1)	CLDN16, PCLN1, HOMG3	3q28	248250: Renal hypomagnesemia 3	Autosomal recessive

**Table 3:** Interventional clinical trials based on the use of citrate supplements with primary or secondary outcomes related to bone health status.

Reference	Study Design; Population	Intervention (Dose/Day) (I) Control (C)	Other Supplements (Dose/Day) and/or Controlled Dietary Intake	Follow Up and Outcomes	BTM Changes (Intragroup)	Changes in BTM and BMD Induced by Intervention (Intergroup)	Conclusion
Dawson-Hughes, 1990 [141]	RCT, controlled vs placebo, double-blind; ≥6 months postmenopausal women (early, <5 years: *n* = 67; late, >5 years: *n* = 169); age ≥ 65 years	I 1: Ca citrate malate (500 mg Ca), *n* = 78 I 2: Ca carbonate (500 mg Ca), *n* = 78 C: Placebo (*n* = 80)	Controlled Ca intake	Baseline, 18, 24, 36 months; BTM (BAP) and BMD	I 1: 24 months ↓ BAP I 2: 24 months ↓ BAP C: 24 months ↓ BAP	BTM I 1 vs. C: 36 months ↓ BAP, related to the Ca intake I 2 vs. C: 36 months ↓ BAP, related to the Ca intake BMD I 1 vs. C: 12, 24 months ↑ only in late postmenopause and Ca intake ≤400 mg/day I 2 vs C: ↓ in both groups	Adequate Ca intake is essential in preventing postmenopausal bone loss; Ca citrate is more effective than Ca carbonate.
Dawson-Hughes, 1997 [142]	RCT, controlled vs. placebo, double-blind; healthy subjects living in a community (176 M/ 213 F); age ≥ 65 years	I: Ca citrate malate (500 mg Ca) & Vit D3 (700 IU), *n* = 187 C: Placebo, *n* = 202	Controlled Ca intake	Baseline, 6, 12, 18, 24, 30, 36 months; BTM (OC, u-NTX) and BMD	I: n.s C: n.s.	BTM I vs. C: 36 months ↓ OC BMD I vs. C: 36 months ↑	Ca and vitamin D supplementation leads to a moderate reduction in bone loss and may substantially reduce the risk of nonvertebral fractures among elderly subjects who live in the community.
* Ruml, 1999 [143]	RCT, controlled vs. placebo; postmenopausal women (90% ≤5 years)	I: Ca citrate (800 mg Ca), *n* = 25 C: Placebo, *n* = 31		Baseline, 12, 24 months BTM (BAP, OC, u-NTX, u- OH proline) and BMD	I: all BTMs are ↓, at unspecified time points	BMD I: 24 months, stable	Ca citrate supplementation averted bone loss and stabilised bone density in early postmenopausal women.
Sellmeyer, 2002 [144]	RCT, controlled vs. placebo, double-blind; ≥2 years postmenopausal women; age I: 65 ± 8 years; C: 63 ± 8 years	I: K citrate (90 mmol), *n* = 26 C: Placebo, *n* = 26	Ca carbonate (500 mg); controlled salt intake	Baseline, 1 months; BTM (OC, u-NTX)	I: n.s. C: 1 month ↓ OC, ↑ u-NTX	BTM I vs. C: 1 month, ↓ u-NTX	K citrate prevents increased bone resorption due to high salt intake.
Dawson-Hughes, 2002 [145]	RCT, controlled vs. placebo, double-blind; healthy subjects (161 M/ 181F); normal BMD; age ≥ 65 years	I: Ca citrate malate (500 mg Ca), *n* = 158 C: Placebo, *n* = 184	Vitamin D3 (700 IU); controlled protein intake	Baseline, 18, 36 months; BTM (OC, u-NTX) and BMD	I: 36 months ↓ u-NTX, related to the protein intake; C: n.s.	BTM I vs C: 36 months, ↓ u-NTX BMD I vs C: 36 month, ↑ related to the protein intake	BMD may be improved by increasing protein intake as long as an adequate intake of Ca and vitamin D is assumed.
Marangella, 2004 [146]	Controlled vs. untreated; postmenopausal women; T score: <−1.0; age: 43–72 years	I: K citrate 37-74 mEq (≈1 mEq/kg), *n* = 30 C: No treatment, *n* = 24	Controlled Ca intake	Baseline, 3 months BTM (BAP, OC, u-OH proline, u-DPD)	I: 3 months ↓ OC, u-OH proline, u-DPD; C: 3 months ↑ OC	not shown	K citrate decreases bone resorption thereby contrasting the potential adverse effects caused by chronic acidemia. The implication for the prevention and treatment of postmenopausal osteoporosis has to be confirmed.
Kenny, 2004 [147]	RCT, crossover, open label, 2 phases; 3 months/phase with a washout period of 2 weeks between phases; postmenopausal women; T score: <−1.0 and >−3.5; age: 73 ± 5 years	I 1: Ca citrate (1000 mg Ca), *n* = 20; I 2: Ca carbonate (1000 mg Ca), *n* = 20	Vitamin D3 (900 IU); controlled Ca intake	Baseline, 1, 3 months (each phase) BTM (BAP, OC, NTX, u-CTX, u-NTX, u-DPD)	I 1: 3 months ↓ NTX, u-CTX, u-NTX, u-DPD I 2: n.s		Ca citrate inhibits bone resorption more than Ca carbonate.
Sakhae, 2005 [139]	RCT, crossover, placebo controlled, double-blind, 4 phases; 2 weeks/phase with a washout period of 2 weeks between phases; postmenopausal women; age: 48–76 years	I 1: K citrate (40 mEq), *n* = 18 I 2: Ca citrate (800 mg), *n* = 18 I 3: K citrate (40 mEq) and Ca citrate (800 mg), *n* = 18 C (1st phase): Placebo, *n* = 18	Rigid diet with fixed content of protein, Ca, P, Na, K and fluids	Baseline and at the end of each phase; BTM (BAP, CTX, OC, u-NTX, u-OH proline)	I 1: n.s I 2: ↓ CTX, u-OH proline I 3: I: ↓ CTX, u-OH proline, u NTX	I 3 vs I 1: ↓ u NTX	In postmenopausal women, combined treatment with K citrate and Ca citrate decreases bone resorption by providing an alkali load and increasing absorbed Ca.
Jehle, 2006 [148]	RCT, controlled; ≥5 years postmenopausal women; T score −1/−4; age: ≤70 years	I: K citrate (30 mEq), *n* = 82 C: KCl (30 mmol), *n* = 79	Ca carbonate (500 mg), Vitamin D3 (400 IU); free, nonvegetarian diet	Baseline, 3, 6, 9, 12 months; BTM (BAP, CTX, OC, u-DPD, u-PD) and BMD	I: 3 months, ↓ u-DPD, u-PD; 6 months, ↑ BAP and ↓ OC, u-DPD, u-PD; 9 months, ↓ u-DPD, u-PD; 12 months, ↑ BAP and ↓ OC, u-DPD, u-PD; C: 3 months, ↓ OC, u-DPD, u-PD; 6 months, ↑ BAP, u-DPD, u-PD and ↓ OC; 9 months, ↑ u-DPD, u-PD and ↓ OC; 12 months, ↑ BAP, u-DPD, u-PD and ↓ OC	BTM I vs C: 3 months, ↓ u-DPD BMD I vs C: 12 months ↑	In postmenopausal women, bone mass can be increased significantly by K citrate. The effect on bone resorption seems to be unrelated to K intake.
Macdonald, 2008 [134]	RCT, controlled vs. placebo, double-blind for I1 e I2; ≥5 years postmenopausal women; age: 49–54 years	I 1: K citrate (55.5 mEq), *n* = 70 I 2: K citrate (18.5 mEq), *n* = 70 I 3: Diet (300 g fruit = 18.5 mEq alkali), *n* = 66 C: Placebo, *n* = 70	Food diary (free nonvegetarian diet)	Baseline, 3, 6, 12, 18, 24 months; BTM (CTX, P1NP, u-DPD) and BMD	I 1: n.s. I 2: n.s. I 3: n.s. C: n.s	BTM I 1, I 2, I 3 vs. C: n.s BMD I 1, I 2, I 3 vs. C: n.s	In healthy postmenopausal women, neither K citrate at 18.5 or 55.6 mEq/d, nor 300 g self-selected fruit and vegetables influenced bone turnover or prevented BMD loss over 2 years.
Thomas, 2008 [149]	RCT, crossover, double-blind, 2 phases; postmenopausal women for 2 to 6 years; age: 50–60 years	I 1: Ca carbonate (1000 mg Ca), *n* = 12 I 2: 2) Ca citrate (500 mg Ca), *n* = 13	Controlled Ca intake	Baseline, 7 days; BTM (CTX)	I 1: 7 days, ↓ CTX I 2: 7 days, ↓ CTX		Ca citrate is at least as effective as Ca carbonate in decreasing PTH and CTX cross-links, at half the dose. All changes are numerically superior after Ca citrate supplementation.
Karp, 2009 [140]	RCT, controlled, 24 h study sessions; women of child-bearing age: 22–30 years	I 1: Ca carbonate (1000 mg Ca), *n* = 12 I 2: Ca citrate (Ca: 1000 mg; citrate: 3145 mg), *n* = 12 I 3: K citrate (K: 57 mEq; citrate: 3145 mg), *n* = 12	4-day diary to estimate food habits before starting the study session; the meals served during each study session were identical	Baseline, 2, 4, 6, 8, 20, 24 h; BTM (BAP, u-NTX) and BMD	I 1: 24 h, ↓ u-NTX I 2: n.s I 3: 24 h, ↓ u-NTX		K citrate supplementation decreases urinary Ca excretion and reduces bone resorption even when the diet is not acidogenic, and reduces the bone resorption marker despite low Ca intake.
Jehle, 2013 [150]	RCT, controlled vs. placebo, double-blind; healthy subjects (79 M/122 F); T score > −2.5; age 65–80 years; women were past the perimenopausal peak turnover	I: K citrate (60 mEq), *n* = 101 C: Placebo, *n* = 100	Ca carbonate (500 mg), Vitamin D3 (400 IU); free nonvegetarian diet	Baseline, 6, 12, 18, 24; BTM (BAP, P1NP, u-NTX) and BMD	I: 6, 12 months, ↓ u-NTX; 18, 24 months, ↑ P1NP C: n.s.	BTM I vs. C: 6 months, ↓ u-NTX BMD I vs. C: 12, 18, 24 months ↑	K citrate administered in a background of vitamin D and Ca supplementation is well tolerated and constitutes an inexpensive intervention to improve BMD and bone microarchitecture in healthy elderly people.
Moseley, 2013 [151]	RCT, controlled vs. placebo, double blind; healthy subjects (17 M/35 F); age ≥ 55 years; women were ≥5 years postmenopause	I 1: K citrate (60 mmol), *n* = 17 I 2: K citrate (90 mmol), *n* = 17 C: Placebo, *n* = 18	Ca citrate (630 mg), Vitamin D3 (400 IU); controlled Ca, Na, P, protein, fat intake	Baseline, 6 months; BTM, (BAP, CTX)		BTM I 1, I2 vs. C: 6 months, ↓ CTX	K citrate decreases markers of bone resorption over 6 months, but a significant improvement in Ca balance is obtained with 90 mmol/day. This dose is well tolerated.
Gregory, 2015 [152]	RCT, controlled vs. placebo, double-blind; ≥2 year postmenopausal women s; T score: <−1.0 > −2.5, or <−2.5 unable to take any other medication; age I: 65.1 ± 5.9 years; C: 66.1 ± 7.1 years	I: K citrate (40 mEq), *n* = 42 C: Placebo, *n* = 41	Ca citrate (630 mg), Vitamin D3 (400 IU); free nonvegetarian diet	Baseline, 1, 3, 6, 12 months; BTM (BAP, OC, P1NP, u-NTX) and BMD	I: 1 month, ↓ P1NP; 3, 6, 12 months, ↓ P1NP, u-NTX C. I: 6 months, ↓ P1NP u-NTX; 12 months, ↓ P1NP	BTM I vs. C: n.s BMD I: 12 months, stable	In postmenopausal osteopenia, K citrate improves the effect of supplementation with Ca citrate and Vitamin D, as proven by the more rapid decrease in BTM levels.
Granchi, 2018 [91]	RCT, controlled vs. placebo, double-blind; ≥5 years postmenopausal women; T score: <−1.0 and >−2.5; age I: 60.8 ± 1.0 years; C: 58.2 ± 1.1 years	I: K citrate (30 mEq), *n* = 20 C: Placebo, *n* = 20	Ca carbonate (500 mg), Vitamin D3 (400 IU); free nonvegetarian diet	Baseline, 3, 6 months; BTM (BAP, CTX, P1NP, TRAcP)	I: 6 months, ↓ BAP, CTX C: 3, 6 months, ↓ BAP, CTX	BTM I vs. C: 6 months ↓ BAP, CTX in subjects with low excretion of K and/or citrate, and/or low urine pH	In postmenopausal osteopenia, K citrate improves the effects of supplementation with Ca carbonate and vitamin D, but only in women with low K and/or citrate excretion and/or low urine pH.

C: control; I: Intervention; RCT: randomised clinical trial; K citrate: potassium citrate; Ca carbonate: calcium carbonate; Ca citrate: calcium citrate; KCl: potassium chloride; Na: sodium; P: phosporus; BMD: bone mineral density; BTM: bone turnover markers; n.a.: not applicable; n.s.: not significant; BAP: bone-specific alkaline phosphatase; BMD: bone mineral density; K: potassium; Ca: calcium; u-DPD: urinary deoxypyridinoline; u-PYR: urinary pyridinoline; u-OH proline: urinary hydroxyproline; OC: osteocalcin; P1NP: amino-terminal propeptide of type 1 procollagen; u-NTX: urinary N-telopeptide of collagen type 1; M: male; F: female; IU: International Units; PTH: parathyroid hormone; ↓ and ↑ show significant decreases and increases, respectively, according to the criteria indicated by the authors. * Partial data collected from the abstract.

**Table 4:** The role of citrate in the pathophysiology and medical management of bone diseases.

Highlights
Citrate is an essential metabolite and plays a pivotal role in maintaining the acid-base balance. Citrate is an essential component of bone, and serves to maintain the integrity of the skeletal nano- and microstructures.
Citrate is produced by osteoblasts but, at the same time, it influences their differentiation and functionality. Bone tissue is the main source of citrate and is therefore a leading actor in maintaining citrate homeostasis. Citrate excretion is a significant biomarker of citrate homeostasis.
Genetic and acquired diseases characterised by an alteration in citrate homeostasis are often accompanied by alterations in the development and/or metabolism of bone tissue. Exogenous supplementation may be a useful tool in treating medical conditions related to poor citrate bioavailability, including bone diseases.

## Abbreviations

acetylCoA: Acetyl Coenzyme A; ATP: Adenosine 5′-Triphosphate; BMD: Bone Mineral Density; BTM: Bone Turnover Marker; CaOx: Calcium-Oxalate; CaP: Calcium-Phosphate; CKD: Chronic Kidney Disease; CTP: mitochondrial Citrate-Transport Protein; dRTA: distal Renal Tubular Acidosis; DXA: Dual-energy X-ray Absorptiometry; GFR: Glomerular Filtration Rate; KDIGO: Kidney Disease Improving Global Outcomes; MSC: Mesenchymal Stem Cell; NaCT: Sodium-dependent Citrate Transporter; NaDC: Sodium-Dicarboxylate; NEAP: Net Endogenous Acid Production; OMIM: Online Mendelian Inheritance in Man; OPRA: Osteoporosis Prospective Risk Assessment; PRAL: Potential Renal Acid Load; PTH: Parathyroid Hormone; RCT: Randomised Clinical Trial; Slc: Solute Carrier; TCA cycle: Tricarboxylic Acid Cycle or Krebs cycle; ZIP1: Zinc Importer Protein 1.

## References

[1] Krebs H.A., Johnson W.A. (1980). The role of citric acid in intermediate metabolism in animal tissues. FEBS Lett..

[2] https://www.genome.jp/kegg-bin/show_pathway?map00020.

[3] Iacobazzi V., Infantino V. (2014). Citrate—New functions for an old metabolite. Biol. Chem..

[4] Mycielska M.E., Milenkovic V.M., Wetzel C.H., Rümmele P., Geissler E.K. (2015). Extracellular Citrate in Health and Disease. Curr. Mol. Med..

[5] Franklin R.B., Chellaiah M., Zou J., Reynolds M.A., Costello L.C. (2014). Evidence that Osteoblasts are Specialized Citrate-producing Cells that Provide the Citrate for Incorporation into the Structure of Bone. Open Bone J..

[6] Dickens F. (1941). The citric acid content of animal tissues, with reference to its occurrence in bone and tumour. Biochem. J..

[7] Haleblian G.E., Leitao V.A., Pierre S.A., Robinson M.R., Albala D.M., Ribeiro A.A., Preminger G.M. (2008). Assessment of citrate concentrations in citrus fruit-based juices and beverages: Implications for management of hypocitraturic nephrolithiasis. J. Endourol..

[8] Penniston K.L., Nakada S.Y., Holmes R.P., Assimos D.G. (2008). Quantitative assessment of citric acid in lemon juice, lime juice, and commercially-available fruit juice products. J. Endourol..

[9] Schuster E., Dunn-Coleman N., Frisvad J.C., Van Dijck P.W. (2002). On the safety of *Aspergillus niger*—A review. Appl. Microbiol. Biotechnol..

[10] Hu W., Li W.J., Yang H.Q., Chen J.H. (2019). Current strategies and future prospects for enhancing microbial production of citric acid. Appl. Microbiol. Biotechnol..

[11] Caudarella R., Vescini F., Buffa A., Stefoni S. (2003). Citrate and mineral metabolism, kidney stones and bone disease. Front. Biosci..

[12] Fegan J., Khan R., Poindexter J., Pak C.Y. (1992). Gastrointestinal citrate absorption in nephrolithiasis. J. Urol..

[13] Pajor A.M. (1999). Sodium-coupled transporters for Krebs cycle intermediates. Annu. Rev. Physiol..

[14] Poerwono H., Higashiyama K., Kubo H., Poernomo A.T., Suharjono I., Sudiana I.K., Indrayanto G., Brittain H.G., Brittain H.G. (2001). Citric acid. Analytical Profiles of Drug Substances and Excipients.

[15] Sakhaee K., Alpern R., Poindexter J., Pak C.Y. (1992). Citraturic response to oral citric acid load. J. Urol..

[16] Zuckerman J.M., Assimos D.G. (2009). Hypocitraturia: Pathophysiology and medical management. Rev. Urol..

[17] Costello L.C., Franklin R.B. (2016). Plasma citrate homeostasis, how it is regulated, and its physiological and clinical implications. An important, but neglected, relationship in medicine. HSOA J. Hum. Endocrinol..

[18] Pak C.Y., Resnick M. (2000). Medical therapy and new approaches to management of urolithiasis. Urol. Clin. N. Am..

[19] Mayo Clinic Medical Laboratories Endocrinology Catalog Bone/Minerals. https//endocrinology.testcatalog.org/show/CITR.

[20] Heller H.J., Sakhaee K., Moe O.W., Pak C.Y. (2002). Etiological role of estrogen status in renal stone formation. J. Urol..

[21] Caudarella R., Vescini F. (2009). Urinary citrate and renal stone disease: The preventive role of alkali citrate treatment. Arch. Ital. Urol..

[22] Melnick J.Z., Preisig P.A., Moe O.W., Srere P., Alpern R.J. (1998). Renal cortical mitochondrial aconitase is regulated in hypo- and hypercitraturia. Kidney Int..

[23] Lerma E.V. Hypocitraturia. Updated 5 October 2015. https://emedicine.medscape.com/article/444968-overview.

[24] Prezioso D., Strazzullo P., Lotti T., Bianchi G., Borghi L., Caione P., Carini M., Caudarella R., Ferraro M., Gambaro G. (2015). Dietary treatment of urinary risk factors for renal stone formation. A review of CLU Working Group. Arch. Ital. Urol..

[25] Dolan E., Sale C. (2019). Protein and bone health across the lifespan. Proc. Nutr. Soc..

[26] Vallés P.G., Batlle D. (2018). Hypokalemic Distal Renal Tubular Acidosis. Adv. Chronic Kidney Dis..

[27] Melnick J.Z., Preisig P.A., Haynes S., Pak C.Y., Sakhaee K., Alpern R.J. (1998). Converting enzyme inhibition causes hypocitraturia independent of acidosis or hypokalemia. Kidney Int..

[28] Warner B.W., LaGrange C.A., Tucker T., Bensalem-Owen M., Pais V.M. (2008). Induction of progressive profound hypocitraturia with increasing doses of topiramate. Urology.

[29] Goraya N., Simoni J., Sager L.N., Madias N.E., Wesson D.E. (2019). Urine citrate excretion as a marker of acid retention in patients with chronic kidney disease without overt metabolic acidosis. Kidney Int..

[30] Shey J., Cameron M.A., Sakhaee K., Moe O.W. (2004). Recurrent calcium nephrolithiasis associated with primary aldosteronism. Am. J. Kidney Dis..

[31] Dey J., Creighton A., Lindberg J.S., Fuselier H.A., Kok D.J., Cole F.E., Hamm L. (2002). Estrogen replacement increased the citrate and calcium excretion rates in postmenopausal women with recurrent urolithiasis. J. Urol..

[32] Brinton R.D. (2008). The healthy cell bias of estrogen action, mitochondrial bioenergetics and neurological implications. Trends Neurosci..

[33] Mai Z., Li X., Jiang C., Liu Y., Chen Y., Wu W., Zeng G. (2019). Comparison of metabolic changes for stone risks in 24-h urine between non- and postmenopausal women. PLoS ONE.

[34] Granchi D., Caudarella R., Ripamonti C., Spinnato P., Bazzocchi A., Torreggiani E., Massa A., Baldini N. (2016). Association between markers of bone loss and urinary lithogenic risk factors in osteopenic postmenopausal women. J. Biol. Regul. Homeost. Agents.

[35] Adeva M.M., Souto G. (2011). Diet-induced metabolic acidosis. Clin. Nutr..

[36] Esche J., Johner S., Shi L., Schönau E., Remer T. (2016). Urinary Citrate, an Index of Acid-Base Status, Predicts Bone Strength in Youths and Fracture Risk in Adult Females. J. Clin. Endocrinol. Metab..

[37] Shea M.K., Dawson-Hughes B. (2018). Association of Urinary Citrate With Acid-Base Status, Bone Resorption, and Calcium Excretion in Older Men and Women. J. Clin. Endocrinol. Metab..

[38] Xie B., Nancollas G.H. (2010). How to control the size and morphology of apatite nanocrystals in bone. Proc. Natl. Acad. Sci. USA.

[39] Dixon T.F., Perkins H.R. (1952). Citric acid and bone metabolism. Biochem. J..

[40] Hu Y.Y., Rawal A., Schmidt-Rohr K. (2010). Strongly bound citrate stabilizes the apatite nanocrystals in bone. Proc. Natl. Acad. Sci. USA.

[41] Mahamid J., Sharir A., Addadi L., Weiner S. (2008). Amorphous calcium phosphate is major component of the forming fin bones of zebrafish. Indications for an amorphous precursor phase. Proc. Natl. Acad. Sci. USA.

[42] Bradt J.H., Mertig M., Teresiak A., Pompe W. (1999). Biomimetic mineralization of collagen by combined fibril assembly and calcium phosphate formation. Chem. Mater..

[43] Lotsari A., Rajasekharan A.K., Halvarsson M., Andersson M. (2018). Transformation of amorphous calcium phosphate to bone-like apatite. Nat. Commun..

[44] Davies E., Müller K.H., Wong W.C., Pickard C.J., Reid D.G., Skepper J.N., Duer M.J. (2014). Citrate bridges between mineral platelets in bone. Proc. Natl. Acad. Sci. USA.

[45] Costello L.C., Chellaiah M., Zou J., Franklin R.B., Reynolds M.A. (2014). The status of citrate in the hydroxyapatite/collagen complex of bone, and Its role in bone formation. J. Regen. Med. Tissue Eng..

[46] Delgado-López J.M., Bertolotti F., Lyngsø J., Pedersen J.S., Cervellino A., Masciocchi N., Guagliardi A. (2017). The synergic role of collagen and citrate in stabilizing amorphous calcium phosphate precursors with platy morphology. Acta Biomater..

[47] Einhorn T.A., Boskey A.L., Gundberg C.M., Vigorita V.J., Devlin V.J., Beyer M.M. (1988). The mineral and mechanical properties of bone in chronic experimental diabetes. J. Orthop. Res..

[48] Tran R.T., Yang J., Ameer G.A. (2015). Citrate-Based Biomaterials and Their Applications in Regenerative Engineering. Annu. Rev. Mater. Res..

[49] Ma C., Gerhard E., Lu D., Yang J. (2018). Citrate chemistry and biology for biomaterials design. Biomaterials.

[50] Qiu H., Yang J., Kodali P., Koh J., Ameer G.A. (2006). A citric acid-based hydroxyapatite composite for orthopedic implants. Biomaterials.

[51] Xie D., Guo J., Mehdizadeh M., Tran R.T., Chen R., Sun D., Qian G., Jin D., Bai X., Yang J. (2015). Development of Injectable Citrate-Based Bioadhesive Bone Implants. J. Mater. Chem. B.

[52] Chung E.J., Sugimoto M.J., Koh J.L., Ameer G.A. (2017). A biodegradable tri-component graft for anterior cruciate ligament reconstruction. J. Tissue Eng. Regen. Med..

[53] Costello L.C., Franklin R.B., Reynolds M.A., Chellaiah M. (2012). The Important Role of Osteoblasts and Citrate Production in Bone Formation: “Osteoblast Citration” as a New Concept for an Old Relationship. Open Bone J..

[54] Costello L.C., Liu Y., Franklin R.B., Kennedy M.C. (1997). Zinc inhibition of mitochondrial aconitase and its importance in citrate metabolism of prostate epithelial cells. J. Biol. Chem..

[55] Franklin R.B., Ma J., Zou J., Guan Z., Kukoyi B.I., Feng P., Costello L.C. (2003). Human ZIP1 is a major zinc uptake transporter for the accumulation of zinc in prostate cells. J. Inorg. Biochem..

[56] Costello L.C., Franklin R.B. (2013). A review of the important central role of altered citrate metabolism during the process of stem cell differentiation. J. Regen. Med. Tissue Eng..

[57] Costello L.C., Chellaiah M.A., Zou J., Reynolds M.A., Franklin R.B. (2015). In vitro BMP2 stimulation of osteoblast citrate production in concert with mineralized bone nodule formation. J. Regen Med. Tissue Eng..

[58] Granchi D., Ochoa G., Leonardi E., Devescovi V., Baglìo S.R., Osaba L., Baldini N., Ciapetti G. (2010). Gene expression patterns related to osteogenic differentiation of bone marrow-derived mesenchymal stem cells during ex vivo expansion. Tissue Eng. Part C Methods.

[59] Fu X., Li Y., Huang T., Yu Z., Ma K., Yang M., Liu Q., Pan H., Wang H., Wang J. (2018). Runx2/Osterix and Zinc Uptake Synergize to Orchestrate Osteogenic Differentiation and Citrate Containing Bone Apatite Formation. Adv. Sci..

[60] Accession Number GSE12267. https://www.ncbi.nlm.nih.gov/geo.

[61] Inoue K., Zhuang L., Ganapathy V. (2002). Human Na+ -coupled citrate transporter: Primary structure, genomic organization, and transport function. Biochem. Biophys. Res. Commun..

[62] Sims N.A., Martin T.J. (2014). Coupling the activities of bone formation and resorption: A multitude of signals within the basic multicellular unit. Bonekey Rep..

[63] Ma C., Tian X., Kim J.P., Xie D., Ao X., Shan D., Lin Q., Hudock M.R., Bai X., Yang J. (2018). Citrate-based materials fuel human stem cells by metabonegenic regulation. Proc. Natl. Acad. Sci. USA.

[64] Granchi D., Torreggiani E., Massa A., Caudarella R., Di Pompo G., Baldini N. (2017). Potassium citrate prevents increased osteoclastogenesis resulting from acidic conditions: Implication for the treatment of postmenopausal bone loss. PLoS ONE.

[65] Arnett T.R. (2010). Acidosis, hypoxia and bone. Arch. Biochem. Biophys..

[66] Lindeman R.D., Tobin J.D., Shock N.W. (1984). Association between blood pressure and the rate of decline in renal function with age. Kidney Int..

[67] Kanis J.A., Johnell O., Oden A., Sembo I., Redlund-Johnell I., Dawson A., De Laet C., Jonsson B. (2000). Long-term risk of osteoporotic fracture in Malmo. Osteoporos Int..

[68] Jassal S.K., von Muhlen D., Barrett-Connor E. (2007). Measures of renal function, BMD, bone loss, and osteoporotic fracture in older adults: The Rancho Bernardo study. J. Bone Min. Res..

[69] (2017). Kidney Disease: Improving Global Outcomes (KDIGO) CKD-MBD Update Work Group. KDIGO 2017 Clinical Practice Guideline Update for the Diagnosis, Evaluation, Prevention, and Treatment of Chronic Kidney Disease-Mineral and Bone Disorder (CKD-MBD). Kidney Int..

[70] Malmgren L., McGuigan F.E., Berglundh S., Westman K., Christensson A., Akesson K. (2015). Declining estimated glomerular filtration rate and its association with mortality and comorbidity over 10 years in elderly women. Nephron.

[71] Malmgren L., McGuigan F.E., Christensson A., Akesson K. (2017). Reduced kidney function is associated with BMD, bone loss and markers of mineral homeostasis in older women: A 10-year longitudinal study. Osteoporos. Int..

[72] Hocher B., Adamski J. (2017). Metabolomics for clinical use and research in chronic kidney disease. Nat. Rev. Nephrol..

[73] Hallan S., Afkarian M., Zelnick L.R., Kestenbaum B., Sharma S., Saito R., Darshi M., Barding G., Raftery D., Ju W. (2017). Metabolomics and Gene Expression Analysis Reveal Down-regulation of the Citric Acid (TCA) Cycle in Non-diabetic CKD Patients. Ebiomedicine.

[74] Kang H.W., Seo S.P., Kim W.T., Kim Y.J., Yun S.J., Lee S.C., Kim W.J. (2014). Effect of renal insufficiency on stone recurrence in patients with urolithiasis. J. Korean Med. Sci..

[75] Krieger N.S., Bushinsky D.A. (2013). The relation between bone and stone formation. Calcif. Tissue Int..

[76] Sakhaee K., Maalouf N.M., Kumar R., Pasch A., Moe O.W. (2011). Nephrolithiasis-associated bone disease: Pathogenesis and treatment options. Kidney Int..

[77] Denburg M.R., Leonard M.B., Haynes K., Tuchman S., Tasian G., Shults J., Copelovitch L. (2014). Risk of fracture in urolithiasis: A population-based cohort study using thehealth improvement network. Clin. J. Am. Soc. Nephrol..

[78] Taylor E.N., Feskanich D., Paik J.M., Curhan G.C. (2016). Nephrolithiasis and Risk of Incident Bone Fracture. J. Urol..

[79] Lucato P., Trevisan C., Stubbs B., Zanforlini B.M., Solmi M., Luchini C., Girotti G., Pizzato S., Manzato E., Sergi G. (2016). Nephrolithiasis, bone mineral density, osteoporosis, and fractures: A systematic review and comparative meta-analysis. Osteoporos. Int..

[80] Khan S.R., Pearle M.S., Robertson W.G., Gambaro G., Canales B.K., Doizi S., Traxer O., Tiselius H.G. (2016). Kidney stones. Nat. Rev. Dis. Primers.

[81] Muntner P., Jones T.M., Hyre A.D., Melamed M.L., Alper A., Raggi P., Leonard M.B. (2009). Association of serum intact parathyroid hormone with lower estimated glomerular filtration rate. Clin. J. Am. Soc. Nephrol..

[82] Han S.G., Oh J., Jeon H.J., Park C., Cho J., Shin D.H. (2019). Kidney Stones and Risk of Osteoporotic Fracture in Chronic Kidney Disease. Sci. Rep..

[83] Arrabal-Polo M.A., Girón-Prieto M.S., Cano-García Mdel C., Poyatos-Andujar A., Quesada Charneco M., Abad-Menor F., Arias-Santiago S., Zuluaga-Gomez A., Arrabal-Martin M. (2015). Retrospective review of serum and urinary lithogenic risk factors in patients with osteoporosis and osteopenia. Urology.

[84] Khosla S., Oursler M.J., Monroe D.G. (2012). Estrogen and the skeleton. Trends Endocrinol. Metab..

[85] Chen H., Wang Y., Dai H., Tian X., Cui Z.K., Chen Z., Hu L., Song Q., Liu A., Zhang Z. (2018). Bone and plasma citrate is reduced in osteoporosis. Bone.

[86] Prochaska M., Taylor E.N., Curhan G. (2018). Menopause and Risk of Kidney Stones. J. Urol..

[87] Drake M.T., Clarke B.L., Lewiecki E.M. (2015). The Pathophysiology and Treatment of Osteoporosis. Clin. Ther..

[88] Rharass T., Lucas S. (2018). Mechanisms in endocrinology: Bone marrow adiposity and bone, a bad romance?. Eur. J. Endocrinol..

[89] Kim J.M., Jeong D., Kang H.K., Jung S.Y., Kang S.S., Min B.M. (2007). Osteoclast precursors display dynamic metabolic shifts toward accelerated glucose metabolism at an early stage of RANKL-stimulated osteoclast differentiation. Cell. Physiol. Biochem..

[90] Lemma S., Sboarina M., Porporato P.E., Zini N., Sonveaux P., Di Pompo G., Baldini N., Avnet S. (2016). Energy metabolism in osteoclast formation and activity. Int. J. Biochem. Cell Biol..

[91] Granchi D., Caudarella R., Ripamonti C., Spinnato P., Bazzocchi A., Massa A., Baldini N. (2018). Potassium Citrate Supplementation Decreases the Biochemical Markers of Bone Loss in a Group of Osteopenic Women: The Results of a Randomized, Double-Blind, Placebo-Controlled Pilot Study. Nutrients.

[92] https://www.omim.org/.

[93] Watanabe T. (2018). Improving outcomes for patients with distal renal tubular acidosis: Recent advances and challenges ahead. Pediatr. Health Med. Ther..

[94] Weinstein D.A., Somers M.J., Wolfsdorf J.I. (2001). Decreased urinary citrate excretion in type 1a glycogen storage disease. J. Pediatr..

[95] Kaiser N., Gautschi M., Bosanska L., Meienberg F., Baumgartner M.R., Spinas G.A., Hochuli M. (2019). Glycemic control and complications in glycogen storage disease type I: Results from the Swiss registry. Mol. Genet. Metab..

[96] Thevenon J., Milh M., Feillet F., St-Onge J., Duffourd Y., Jugé C., Roubertie A., Héron D., Mignot C., Raffo E. (2014). Mutations in SLC13A5 cause autosomal-recessive epileptic encephalopathy with seizure onset in the first days of life. Am. J. Hum. Genet..

[97] Irizarry A.R., Yan G., Zeng Q., Lucchesi J., Hamang M.J., Ma Y.L., Rong J.X. (2017). Defective enamel and bone development in sodium-dependent citrate transporter (NaCT) Slc13a5 deficient mice. PLoS ONE.

[98] Díaz M., García C., Sebastiani G., de Zegher F., López-Bermejo A., Ibáñez L. (2017). Placental and cord blood methylation of genes involved in energy homeostasis: Association with fetal growth and neonatal body composition. Diabetes.

[99] Cunha T.D.S., Heilberg I.P. (2018). Bartter syndrome: Causes, diagnosis, and treatment. Int. J. Nephrol. Renov. Dis..

[100] Simon D.B., Karet F.E., Hamdan J.M., DiPietro A., Sanjad S.A., Lifton R.P. (1996). Bartter’s syndrome, hypokalaemic alkalosis with hypercalciuria, is caused by mutations in the Na-K-2Cl cotransporter NKCC2. Nat. Genet..

[101] International Collaborative Study Group for Bartter-like Syndromes (1997). Mutations in the gene encoding the inwardly-rectifying renal potassium channel, ROMK, cause the antenatal variant of Bartter syndrome: Evidence for genetic heterogeneity. Hum. Molec. Genet..

[102] Gross I., Siedner-Weintraub Y., Simckes A., Gillis D. (2015). Antenatal Bartter syndrome presenting as hyperparathyroidism with hypercalcemia and hypercalciuria: A case report and review. J. Pediatr. Endocrinol. Metab..

[103] Li D., Tian L., Hou C., Kim C.E., Hakonarson H., Levine M.A. (2016). Association of Mutations in SLC12A1 Encoding the NKCC2 Cotransporter with Neonatal Primary Hyperparathyroidism. J. Clin. Endocrinol. Metab..

[104] Hou J. (2016). Claudins and mineral metabolism. Curr. Opin. Nephrol. Hypertens..

[105] Thorleifsson G., Holm H., Edvardsson V., Walters G.B., Styrkarsdottir U., Gudbjartsson D.F., Sulem P., Halldorsson B.V., de Vegt F., d’Ancona F.C. (2009). Sequence variants in the CLDN14 gene associate with kidney stones and bone mineral density. Nat. Genet..

[106] Claverie-Martin F. (2015). Familial hypomagnesaemia with hypercalciuria and nephrocalcinosis, clinical and molecular characteristics. Clin. Kidney J..

[107] Bardet C., Courson F., Wu Y., Khaddam M., Salmon B., Ribes S., Thumfart J., Yamaguti P.M., Rochefort G.Y., Figueres M.L. (2016). Claudin-16 Deficiency Impairs Tight Junction Function in Ameloblasts, Leading to Abnormal Enamel Formation. J. Bone Min. Res..

[108] Pajor A.M. (2014). Sodium-coupled dicarboxylate and citrate transporters from the SLC13 family. Pflug. Arch.

[109] Okamoto N., Aruga S., Matsuzaki S., Takahashi S., Matsushita K., Kitamura T. (2007). Associations between renal sodium-citrate cotransporter (hNaDC-1) gene polymorphism and urinary citrate excretion in recurrent renal calcium stone formers and normal controls. Int. J. Urol..

[110] Pajor A.M., Sun N.N. (2010). Single nucleotide polymorphisms in the human Na+-dicarboxylate cotransporter affect transport activity and protein expression. Am. J. Physiol. Ren. Physiol..

[111] Catalina-Rodriguez O., Kolukula V.K., Tomita Y., Preet A., Palmieri F., Wellstein A., Byers S., Giaccia A.J., Glasgow E., Albanese C. (2012). The mitochondrial citrate transporter, CIC, is essential for mitochondrial homeostasis. Oncotarget.

[112] Cosso R., Falchetti A. (2018). Mitochondriopathies and bone health. J. Tre. Biol. Res..

[113] Brommage R., Liu J., Hansen G.M., Kirkpatrick L.L., Potter D.G., Sands A.T., Zambrowicz B., Powell D.R., Vogel P. (2014). High-throughput screening of mouse gene knockouts identifies established and novel skeletal phenotypes. Bone Res..

[114] Lorentzon M., Branco J., Brandi M.L., Bruyère O., Chapurlat R., Cooper C., Cortet B., Diez-Perez A., Ferrari S., Gasparik A. (2019). Algorithm for the Use of Biochemical Markers of Bone Turnover in the Diagnosis, Assessment and Follow-Up of Treatment for Osteoporosis. Adv. Ther..

[115] Kanis J.A., Cooper C., Rizzoli R., Reginster J.Y., Scientific Advisory Board of the European Society for Clinical and Economic Aspects of Osteoporosis (ESCEO) and the Committees of Scientific Advisors and National Societies of the International Osteoporosis Foundation (IOF) (2019). European guidance for the diagnosis and management of osteoporosis in postmenopausal women. Osteoporos. Int..

[116] Ripamonti C., Lisi L., Buffa A., Gnudi S., Caudarella R. (2018). The Trabecular Bone Score Predicts Spine Fragility Fractures in Postmenopausal Caucasian Women Without Osteoporosis Independently of Bone Mineral Density. Med. Arch..

[117] Damasiewicz M.J., Nickolas T.L. (2018). Rethinking Bone Disease in Kidney Disease. JBMR Plus.

[118] Moe S., Drüeke T., Cunningham J., Goodman W., Martin K., Olgaard K., Ott S., Sprague S., Lameire N., Eknoyan G. (2006). Kidney Disease: Improving Global Outcomes (KDIGO). Definition, evaluation, and classification of renal osteodystrophy: A position statement from Kidney Disease: Improving Global Outcomes (KDIGO). Kidney Int..

[119] Jorgetti V., Drüeke T.B., Ott S.M. (2016). Role of proton receptor OGR1 in bone response to metabolic acidosis?. Kidney Int..

[120] Wachman A., Bernstein D.S. (1968). Diet and osteoporosis. Lancet.

[121] Nicoll R., McLaren Howard J. (2014). The acid-ash hypothesis revisited: A reassessment of the impact of dietary acidity on bone. J. Bone Min. Metab..

[122] Frassetto L.A., Todd K.M., Morris R.C., Sebastian A. (1998). Estimation of net endogenous noncarbonic acidproduction in humans from diet potassium and protein contents. Am. J. Clin. Nutr..

[123] Remer T., Manz F. (1994). Estimation of the renal net acid excretion by adults consuming diets containing variable amounts of protein. Am. J. Clin. Nutr..

[124] Pachaly M.A., Baena C.P., Buiar A.C., de Fraga F.S., Carvalho M. (2016). Effects of non-pharmacological interventions on urinary citrate levels: A systematic review and meta-analysis. Nephrol. Dial. Transpl..

[125] Frassetto L., Banerjee T., Powe N., Sebastian A. (2018). Acid Balance, Dietary Acid Load, and Bone Effects-A Controversial Subject. Nutrients.

[126] Fenton T.R., Lyon A.W., Eliasziw M., Tough S.C., Hanley D.A. (2009). Meta-analysis of the effect of the acid-ash hypothesis of osteoporosis on calcium balance. J. Bone Min. Res..

[127] Bonjour J.P. (2013). Nutritional disturbance in acid-base balance and osteoporosis: A hypothesis that disregards the essential homeostatic role of the kidney. Br. J. Nutr..

[128] Carnauba R.A., Baptistella A.B., Paschoal V., Hübscher G.H. (2017). Diet-Induced Low-Grade Metabolic Acidosis and Clinical Outcomes: A Review. Nutrients.

[129] Hirschfeld H.P., Kinsella R., Duque G. (2017). Osteosarcopenia: Where bone, muscle, and fat collide. Osteoporos. Int..

[130] Cases A., Cigarrán-Guldrís S., Mas S., Gonzalez-Parra E. (2019). Vegetable-Based Diets for Chronic Kidney Disease? It Is Time to Reconsider. Nutrients.

[131] Domrongkitchaiporn S., Stitchantrakul W., Kochakarn W. (2006). Causes of hypocitraturia in recurrent calcium stone formers: Focusing on urinary potassium excretion. Am. J. Kidney Dis..

[132] Odvina C.V. (2006). Comparative value of orange juice versus lemonade in reducing stone-forming risk. Clin. J. Am. Soc. Nephrol..

[133] Goldfarb D.S., Asplin J.R. (2001). Effect of grapefruit juice on urinary lithogenicity. J. Urol..

[134] Macdonald H.M., Black A.J., Aucott L., Duthie G., Duthie S., Sandison R., Hardcastle A.C., Lanham New S.A., Fraser W.D., Reid D.M. (2008). Effect of potassium citrate supplementation or increased fruit and vegetable intake on bone metabolism in healthy postmenopausal women: A randomized controlled trial. Am. J. Clin. Nutr..

[135] Phillips R., Hanchanale V.S., Myatt A., Somani B., Nabi G., Biyani C.S. (2015). Citrate salts for preventing and treating calcium containing kidney stones in adults. Cochrane Database Syst. Rev..

[136] Kern A., Grimsby G., Mayo H., Baker L.A. (2017). Medical and dietary interventions for preventing recurrent urinary stones in children. Cochrane Database Syst. Rev..

[137] Lencel P., Magne D. (2011). Inflammaging: The driving force in osteoporosis?. Med. Hypotheses.

[138] Lambert H., Frassetto L., Moore J.B., Torgerson D., Gannon R., Burckhardt P., Lanham-New S. (2015). The effect of supplementation with alkaline potassium salts on bone metabolism: A meta-analysis. Osteoporos. Int..

[139] Sakhaee K., Maalouf N.M., Abrams S.A., Pak C.Y. (2005). Effects of potassium alkali and calcium supplementation on bone turnover in postmenopausal women. J. Clin. Endocrinol. Metab..

[140] Karp H.J., Ketola M.E., Lamberg-Allardt C.J. (2009). Acute effects of calcium carbonate, calcium citrate and potassium citrate on markers of calcium and bone metabolism in young women. Br. J. Nutr..

[141] Dawson-Hughes B., Dallal G.E., Krall E.A., Sadowski L., Sahyoun N., Tannenbaum S. (1990). A Controlled Trial of the Effect of Calcium Supplementation on Bone Density in Postmenopausal Women. N. Engl. J. Med..

[142] Dawson-Hughes B., Harris S.S., Krall E.A., Dallal G.E. (1997). Effect of Calcium and Vitamin D Supplementation on Bone Density in Men and Women 65 Years of Age Or Older. N. Engl. J. Med..

[143] Ruml L.A., Sakhaee K., Peterson R., Adams-Huet B., Pak C.Y. (1999). The Effect of Calcium Citrate on Bone Density in the Early and Mid-Postmenopausal Period: A Randomized Placebo-Controlled Study. Am. J. Ther..

[144] Sellmeyer D.E., Schloetter M., Sebastian A. (2002). Potassium Citrate Prevents Increased Urine Calcium Excretion and Bone Resorption Induced by a High Sodium Chloride Diet. J. Clin. Endocrinol. Metab..

[145] Dawson-Hughes B., Harris S.S. (2002). Calcium Intake Influences the Association of Protein Intake with Rates of Bone Loss in Elderly Men and Women. Am. J. Clin. Nutr..

[146] Marangella M., Di Stefano M., Casalis S., Berutti S., D’Amelio P., Isaia G.C. (2004). Effects of potassium citrate supplementation on bone metabolism. Calcif. Tissue Int..

[147] Kenny A.M., Prestwood K.M., Biskup B., Robbins B., Zayas E., Kleppinger A., Burleson J.A., Raisz L.G. (2004). Comparison of the Effects of Calcium Loading with Calcium Citrate Or Calcium Carbonate on Bone Turnover in Postmenopausal Women. Osteoporos. Int..

[148] Jehle S., Zanetti A., Muser J., Hulter H.N., Krapf R. (2006). Partial Neutralization of the Acidogenic Western Diet with Potassium Citrate Increases Bone Mass in Postmenopausal Women with Osteopenia. J. Am. Soc. Nephrol..

[149] Thomas S.D., Need A.G., Tucker G., Slobodian P., O’Loughlin P.D., Nordin B.E. (2008). Suppression of parathyroid hormone and bone resorption by calcium carbonate and calcium citrate in postmenopausal women. Calcif. Tissue Int..

[150] Jehle S., Hulter H.N., Krapf R. (2013). Effect of potassium citrate on bone density, microarchitecture, and fracture risk in healthy older adults without osteoporosis: A randomized controlled trial. J. Clin. Endocrinol. Metab..

[151] Moseley K.F., Weaver C.M., Appel L., Sebastian A., Sellmeyer D.E. (2013). Potassium Citrate Supplementation Results in Sustained Improvement in Calcium Balance in Older Men and Women. J. Bone Min. Res..

[152] Gregory N.S., Kumar R., Stein E.M., Alexander E., Christos P., Bockman R.S., Rodman J.S. (2015). Potassium Citrate Decreases Bone Resorption in Postmenopausal Women with Osteopenia: A Randomized, Double-Blind Clinical Trial. Endocr. Pr..

[153] Anderson N.M., Mucka P., Kern J.G., Feng H. (2018). The emerging role and targetability of the TCA cycle in cancer metabolism. Protein Cell.

[154] Sathiyakumar V., Kapoor K., Jones S.R., Banach M., Martin S.S., Toth P.P. (2018). Novel Therapeutic Targets for Managing Dyslipidemia. Trends Pharm. Sci..

[155] Ou Y., Liu Z., Li S., Zhu X., Lin Y., Han J., Duan Z., Jia L., Gui B. (2017). Citrate attenuates vascular calcification in chronic renal failure rats. APMIS.

[156] Michalczuk M., Urban B., Porowski T., Wasilewska A., Bakunowicz-Łazarczyk A. (2018). Citrate usage in the leading causes of blindness: New possibilities for the old metabolite. Metabolomics.

